# Advances in Physicochemical and Biological Treatment of Textile Wastewater: An Applied Review of Methods, Technologies, and Costs

**DOI:** 10.1002/wer.70490

**Published:** 2026-07-18

**Authors:** Adedapo O. Adeola, Stephanie Gora

**Affiliations:** ^1^ Civil Engineering, Lassonde School of Engineering York University Toronto ON Canada

**Keywords:** advanced oxidation processes, environmental pollution, microbial fuel cells, physicochemical treatment, textile wastewater

## Abstract

The textile industry is a major source of water pollution worldwide, including many countries in the Global South. Effluents from washing, bleaching, and dyeing contain dyes, heavy metals, surfactants, and other toxic compounds that threaten ecosystems and human health. Although wastewater treatment plants (WWTPs) reduce pollution, they face high costs, sludge generation, and limited removal of persistent contaminants. To improve performance, physicochemical, biological, and hybrid technologies have been developed. Coagulation–flocculation and adsorption are cost‐effective but produce secondary waste. Biological systems such as enzymatic treatment and microbial fuel cells enable pollutant removal with potential energy recovery, though scale‐up remains limited. Emerging methods such as nanofiltration, nanobubble technology, and electrochemical advanced oxidation processes (AOPs) provide higher efficiencies but face material stability issues. This review examines current technologies, their mechanisms, limitations, and prospects for achieving effective, economical, and environmentally sustainable textile wastewater management, supported by an assessment of effluent quality compliance and techno‐economic feasibility. The ultimate goals of the review are to identify major research gaps and treatment processes that are effective, scalable, and implementable in real textile wastewater treatment installations and to propose concrete research directions that will enable the translation of promising laboratory results to full‐scale implementation for textile wastewater treatment.

## Summary

- Textile effluents contain toxic dyes, metals, and surfactants harming ecosystems.
- High costs and stability issues hinder photocatalysis scale‐up and adoption.
- Adsorption is viable for treatment, though secondary pollution remains a concern.
- Hybrid treatment methods improve efficiency for sustainable wastewater reuse.
- Technoeconomic analysis guides feasible, scalable treatment technologies.

## Graphical abstract

Major textile wastewater treatment technologies, including physicochemical (adsorption, membrane filtration, coagulation–flocculation, advanced oxidation), biological (algal–bacterial systems, fungal treatment, anaerobic digestion, microbial fuel cells), and hybrid approaches. It also highlights key evaluation factors such as cost, efficiency, energy demand, regeneration challenges, and catalyst stability.

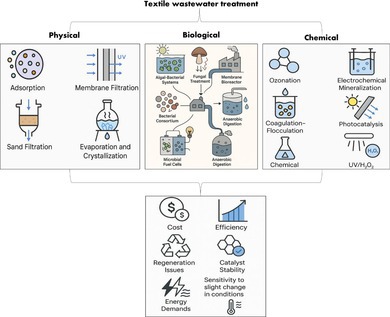

## 1 Introduction

**FIGURE 1:**
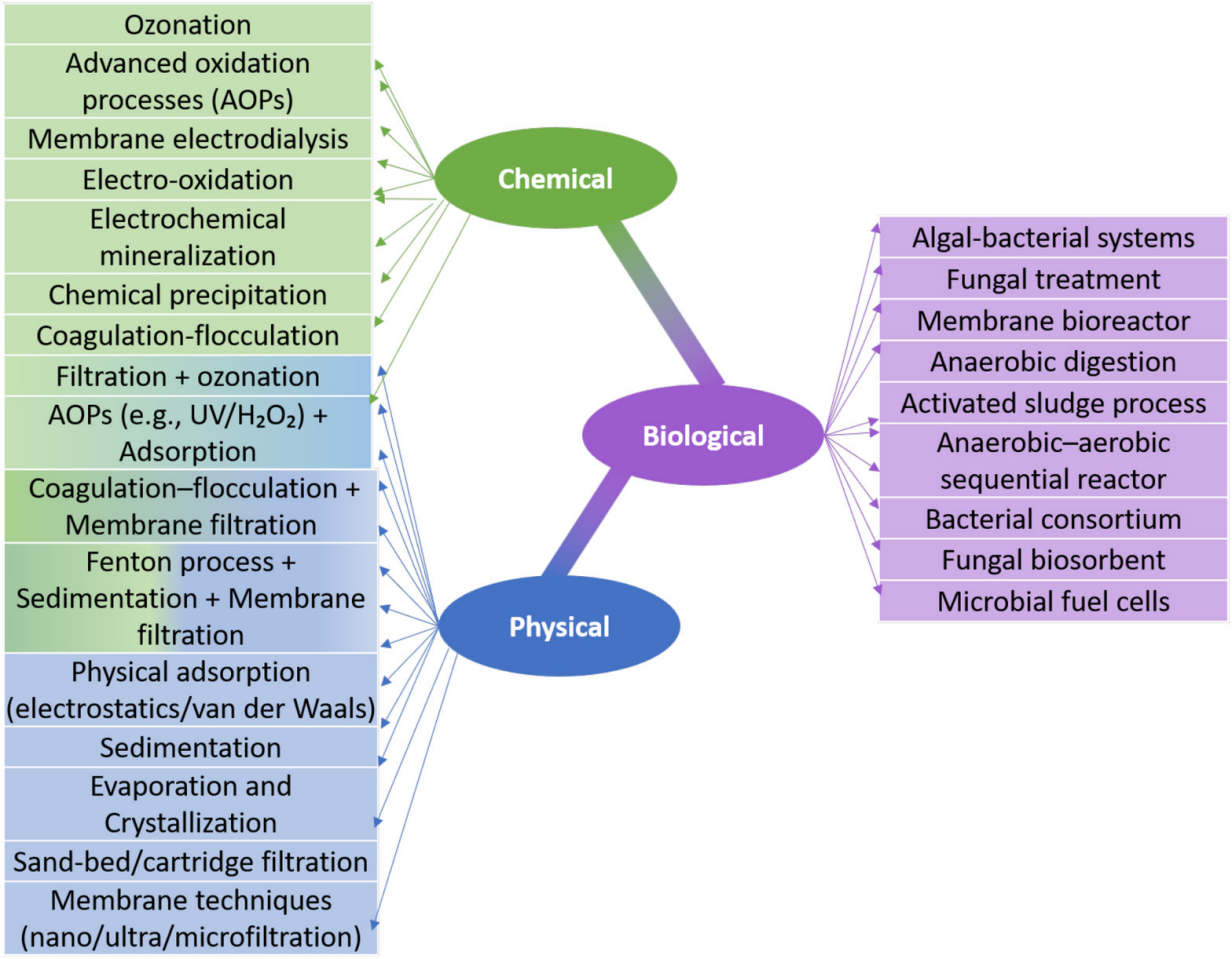
Types of physical, chemical, biological, and hybrid methods that have been reported for textile wastewater treatment in the peer‐reviewed literature.

The textile industry is one of the largest globally with businesses worth billions of dollars worldwide (Fernandez‐Stark et al. 2022). Nonetheless, textile manufacturing remains one of the world's greatest sources of water pollution via its multiple chemical‐intensive processes that include washing, scouring, bleaching, dyeing, and finishing operations (Halepoto et al. 2022; Rahman et al. 2022). Wastewater from these operations containing hazardous and toxic materials is released into the water bodies by textile mills, thus putting both humans and aquatic flora and fauna at risk (Lellis et al. 2019). Wastewater treatment plants (WWTPs) are vital for mitigating pollution and enabling water reuse, especially in the textile industry, where large volumes of wastewater containing dyes and other harmful chemicals are produced (Singh et al. 2023). WWTPs remove contaminants through multi‐stage processes that facilitate safe disposal or reuse of treated wastewater, however, challenges exist, such as increased capital and operational costs required to address emerging contaminants (e.g., synthetic dyes). Textile effluents may contain dyes, heavy metals, and organic/inorganic chemicals originating from dyeing, printing, and finishing processes and require tailored treatment approaches. Several textile wastewater treatment processes have been explored in the academic literature (Fernandes et al. 2024; Halepoto et al. 2022). These methods can be categorized into either physical, chemical, or biological techniques (Figure 1). More recently, several hybrid techniques that combine two or more techniques have been developed at the laboratory scale. In practice, many real world installations employ multi‐step treatment processes and/or send their wastewater to municipal WWTPs (Lu et al. 2010; Ranganathan et al. 2007).

**FIGURE 2:**
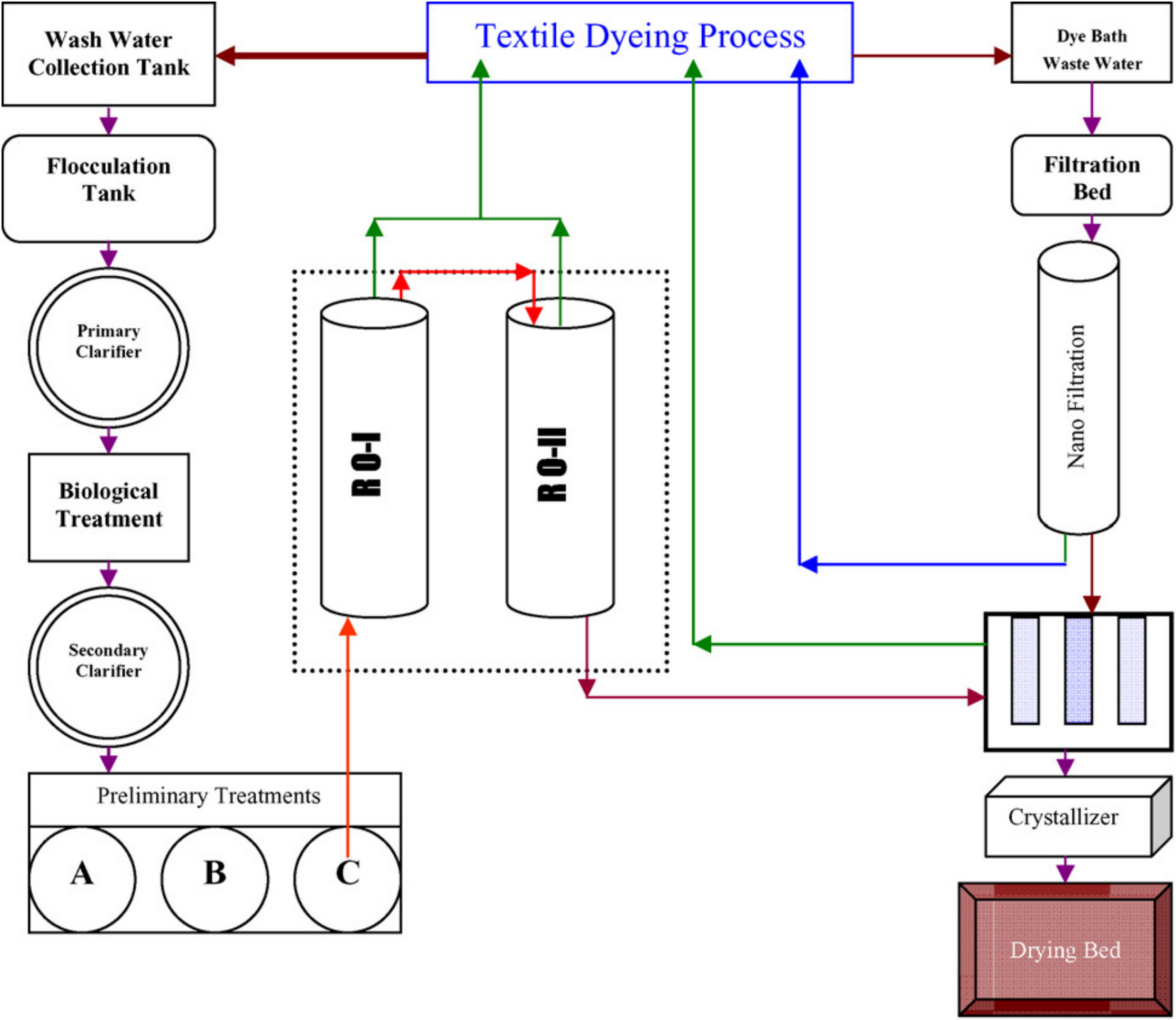
Representative schematic of advanced treatment technologies for the recycling of textile dyeing wastewater (Ranganathan et al. 2007).

An example of a dye‐effluent treatment plant is depicted in Figure 2. The process begins by separately collecting dye‐bath and wash‐water effluents (Ranganathan et al. 2007). Dye bath wastewater is first treated via sand filtration, followed by nanofiltration (NF). The NF permeate is reused in the dyeing process, while the 20%–30% reject is sent to a multi‐effect evaporator (MEE) or solar evaporation pond for concentration and recovery. Simultaneously, wash waters undergo sequential physicochemical and biological treatments before being processed through a two‐stage reverse osmosis (RO) system (Figure 2). The RO permeate is recycled, while the 15%–20% reject is either subjected to NF for salt recovery or forwarded to evaporators. Final rejects from the NF systems are also channeled to the MEE, from which condensed water is recovered and minimal final effluent (2%–3%) is disposed of, typically via solar evaporation, with solid residues managed appropriately. This case study offers insights into real‐world treatment of textile wastewater, which more often than not involves a multi‐process involving several physicochemical and biological treatment techniques.

## 2 Review Methodology

This review examines physicochemical, biological, and emerging treatment technologies for textile wastewater treatment, with particular emphasis on adsorption, membrane filtration, AOPs, photocatalysis, and bioremediation. Literature retrieval was conducted using Google Scholar, Scopus, Web of Science, and the York University Library databases. The search was performed between January 2025 and June 2026 and included peer‐reviewed journal articles published primarily between 2000 and 2026, although earlier studies were included where relevant. The search strategy employed combinations of keywords and Boolean operators, including: “textile wastewater treatment”, “textile effluent”, “synthetic dye removal”, “emerging contaminants”, “dyes”, “inorganic salts”, “antimicrobials”, “heavy metals”, “organometallics”, “flame retardants”, “per‐ and polyfluoroalkyl substances (PFAS)”, “phthalates”, “polycyclic aromatic hydrocarbons (PAHs)”, “aromatic amines”, “UV absorbers”, “biochemical oxygen demand (BOD)”, “chemical oxygen demand (COD)”, “advanced oxidation processes”, “photocatalysis”, “adsorption”, “nanofiltration”, “microfiltration”, “ultrafiltration”, “reverse osmosis”, “electrodialysis”, “membrane distillation”, and “bioremediation”.

Studies were included if they: (i) evaluated treatment technologies for real‐world or synthetic textile wastewater; (ii) reported quantitative performance metrics such as contaminant removal efficiency and/or adsorption capacity, treatment parameters, energy consumption, or operational cost; (iii) contained sufficient experimental or operational details for comparison; and (iv) were published in peer‐reviewed journals. Studies were excluded if they lacked quantitative treatment data, focused solely on analytical detection methods, or provided insufficient methodological information. Following literature retrieval, duplicate records were removed, and titles, abstracts, and full texts were screened for relevance. Data extracted from eligible studies included treatment technology, target contaminants, operational conditions, treatment efficiency, energy requirements (where available), cost information, and scale of implementation. To ensure consistency, studies were classified according to their reported scale of operation as follows: laboratory scale (bench‐scale experiments conducted under controlled conditions), semi‐pilot scale (intermediate systems incorporating partial process integration), pilot scale (pre‐commercial systems designed to simulate industrial operating conditions), and full scale (commercial or industrial treatment systems operating under real‐world conditions).

The quality of included studies was assessed based on the completeness of experimental descriptions, reporting of operational parameters, reproducibility of methods, and availability of quantitative performance metrics. Comparative analyses of treatment performance were conducted using reported contaminant removal efficiencies, adsorption capacities, water recovery rates, and effluent quality indicators, including COD, BOD, color, nutrients, salts, and emerging contaminants. Because of substantial variability in wastewater composition, operating conditions, and reporting formats across studies, a formal meta‐analysis was not performed. Instead, a qualitative and comparative synthesis approach was adopted to identify performance trends, technological limitations, scalability challenges, and research gaps.

A subset of studies containing economic information was further evaluated using techno‐economic considerations, including reported capital costs, operating costs, energy consumption, chemical requirements, maintenance demands, and scalability potential. To improve comparability among studies conducted in different years, reported cost data were adjusted to January 2026 values, where sufficient information was available, using the Consumer Price Index (CPI). Cost normalization was performed according to the following Equation (5):

(1) $$\text{Adjusted Cost}=\text{Historical Cost}\times \left({\mathrm{CPI}}_{\left(2026\right)}/{\mathrm{CPI}}_{\left(\text{original year}\right)}\right)$$

This approach enabled inflation‐adjusted comparisons of treatment costs. It is noteworthy to mention that regional differences in labor, energy prices, chemical costs, and regulatory requirements may still influence economic outcomes. Given the limited availability of full‐scale economic data in the peer‐reviewed literature, these assessments are intended to provide indicative comparisons rather than definitive cost estimates. The review also evaluates treatment technologies in the context of compliance with Zero Discharge of Hazardous Chemicals (ZDHC) guidelines and their potential contribution toward sustainable textile wastewater management.

## 3 Nature of Textile Wastewater/Effluents and Environmental Implications

Among the various chemicals present, synthetic dyes are the most visible and challenging. Reactive, disperse, direct, and vat dyes are widely used in textile manufacturing, and a significant fraction may remain unfixed during dyeing operations and subsequently enter wastewater streams (Akter et al. 2023). Many textile dyes exhibit high chemical stability and resistance to biodegradation, particularly azo dyes, resulting in their persistence in aquatic environments. In aquatic systems, dye‐contaminated effluents reduce light penetration, interfere with photosynthetic activity, impair dissolved oxygen transfer, and may exert toxic effects on organisms (Berradi et al. 2019; Ismail et al. 2019).

Inorganic salts, particularly sodium chloride (NaCl) and sodium sulfate (Na_2_SO_4_), are extensively used during dyeing processes to enhance dye fixation and fabric uptake (Yildirim et al. 2023). Residual salts discharged with wastewater contribute significantly to the high conductivity and salinity of textile effluents, posing challenges for biological treatment processes and limiting opportunities for water reuse (Ru et al. 2018). In addition to the prominent pollutants, textile wastewater may contain a range of contaminants of emerging concern (CECs), including per‐ and polyfluoroalkyl substances (PFAS), phthalates, flame retardants, polycyclic aromatic hydrocarbons (PAHs), antimicrobial agents, and ultraviolet (UV) absorbers (Wang, Jiang, and Gao 2022; Xiao et al. 2024). These compounds are commonly associated with textile finishing operations and functional fabric production. Their persistence, potential for bioaccumulation, and resistance to conventional treatment technologies have raised increasing environmental and regulatory concerns (Cantoni et al. 2024; Hou et al. 2021). Textile effluents also typically exhibit elevated COD and BOD levels due to the presence of dyes, surfactants, organic additives, and processing chemicals. High organic loading can deplete dissolved oxygen in receiving waters, adversely affecting aquatic ecosystems and reducing water quality (Abid et al. 2024). Furthermore, wastewater discharged at elevated temperatures or extreme pH values may increase ecological impacts and hinder downstream treatment processes (Abu Bakar et al. 2020).

The diverse chemical composition, variability, and persistence of contaminants in textile wastewater necessitate the development and implementation of effective treatment technologies capable of achieving regulatory compliance and supporting sustainable water management. Consequently, a combination of physical, chemical, biological, and hybrid treatment approaches has been explored to address the limitations of conventional treatment systems and improve the removal of both prominent pollutants and emerging contaminants.

## 4 Treatment of Textile Wastewater

Over the past decades, a variety of physical, chemical, and biological processes have been developed and used in textile wastewater treatment installations, often in combination, to address treatment challenges and enhance efficiency. Public health concerns and the growing demand for sustainable water management have driven the advancement of textile wastewater treatment technologies. Integrated treatment installations that combine conventional methods with advanced processes such as membrane filtration, advanced oxidation, and hybrid biological–chemical systems have shown promise in achieving near‐complete removal of color, organic load, and emerging contaminants while enabling water reuse (Xiangyu et al. 2025). Nevertheless, issues such as high energy demand, sludge generation, membrane fouling, and the incomplete mineralization of recalcitrant pollutants remain persistent obstacles to large‐scale adoption (Xue et al. 2025). The following sections review current approaches to full‐scale treatment and examine the major categories of treatment technologies: physical, chemical, and biological, highlighting their potential role in advancing sustainable wastewater management in the textile industry.

### 4.1 Current Approaches to Full‐Scale Treatment of Textile Wastewater

Information about full‐scale textile wastewater treatment is very limited in the peer‐reviewed academic literature; however, a subset of studies has described real textile wastewater treatment systems. These processes are often complex, combining a series of physical, chemical, and sometimes biological treatment processes. For example, Jia, Farid, et al. (2023) investigated three full‐scale textile dyeing WWTPs in China to evaluate the fate of 27 legacy and emerging PFAS. Influent concentrations of PFASs ranged from 630 to 4268 ng/L, with effluents containing 436–755 ng/L and sludge 91.5–1182 μg/kg dry weight (Jia, Shan, et al. 2023). The plants displayed distinct PFAS profiles, with one dominated by legacy perfluorocarboxylic acids and the others by emerging compounds, reflecting differences in industrial inputs. Conventional processes such as primary treatment and biological degradation showed limited PFAS removal and, in some cases, facilitated the formation of terminal perfluoroalkyl acids (PFAAs) from precursors. RO achieved > 90% removal but generated PFAS‐rich concentrate, while total oxidizable precursor assays revealed that oxidative steps could increase PFAS levels by 2.3–4.1‐fold through precursor transformation (Jia, Shan, et al. 2023). These findings highlight the persistence of PFAS in textile effluents, the limited effectiveness of conventional textile wastewater treatment, and the critical role of textile wastewater sludge and concentrate streams as PFAS reservoirs.

Lu et al. (2010) and Ranganathan et al. (2007) each evaluated textile wastewater treatment installations in India that combine conventional chemical, biological, or physical pretreatment with membrane filtration, demonstrating that RO is highly effective at removing a broad suite of contaminants (Lu et al. 2010; Ranganathan et al. 2007). Lu et al. (2010) examined treatment installations incorporating primary and secondary treatment in addition to RO, reporting that COD, color, and turbidity removals all exceeded 90%, enabling reuse or near‐zero‐liquid discharge in some cases. Ranganathan et al. (2007) examined coagulation/flocculation, biological oxidation, and filtration, showing that RO substantially reduced dissolved solids, color, and organic load relative to pretreatment alone, although synthetic and recalcitrant compounds were more difficult to remove. Together, these studies underscore that while pretreatment is necessary to protect membranes and reduce contaminant load, RO acts as the critical polishing step for achieving high‐quality effluents in textile wastewater treatment.

In a study by Paździor et al. (2017), two installation systems in Poland were employed for the treatment of textile wastewater. A sequence batch reactor (SBR) with a working volume of ≈ 1.5 dm^3^ and a horizontal continuous flow bioreactor (HCFB) with a working volume of ≈12 dm^3^ (Paździor et al. 2017). Both were operated at ambient temperature (reported at ~20°C–25°C, no heating), under aerobic conditions, with hydraulic retention times sufficient to allow measurable biodegradation (exact HRTs varied depending on experimental run), and biomass concentrations appropriate for small lab‐scale reactors. The SBR was operated in batch mode, allowing fill, react/aerate, settle, and decant cycles, while the HCFB was run continuously with a steady flow of wastewater through the bioreactor. The combined treatment schemes (e.g., biodegradation then ozonation or biodegradation‐ozonation‐biodegradation) were evaluated for removal of organic carbon (COD, TOC) and acute toxicity (Microtox‐EC50), with the two‐stage biodegradation‐ozonation scheme in HCFB achieving up to ~98% toxicity reduction (Paździor et al. 2017).

### 4.2 Representation of Full‐Scale Systems in Peer‐Reviewed Laboratory Studies

The complexity of textile wastewater treatment in full scale applications has rarely been reflected in the peer‐reviewed experimental literature, which has instead focused on the removal of single contaminants, often dyes, from pure water matrices by various physical, chemical, and biological methods. Sections 5, 6, and 7 provide up‐to‐date syntheses of past research into textile wastewater treatment by treatment processes in these three major categories.

## 5 Physical Processes for the Treatment of Textile Wastewater

Physical treatment processes primarily remove contaminants through physical separation, transfer, or retention mechanisms rather than direct chemical transformation of the pollutants. Examples of physical treatment methods include sedimentation, filtration, degasification, and adsorption. While these processes do not intentionally degrade contaminants, they may alter contaminant mobility, bioavailability, or distribution between environmental phases through interactions with treatment media or particulate matter. However, there are several drawbacks to physical processes, including selectivity concerns, limited capacity, and time consumption (Saravanan et al. 2021; Sharma and Bhattacharya 2017). To achieve improved water treatment efficiency and size‐based selectivity for contaminants, advanced methods such as RO, NF, ion exchange, ultrafiltration (UF), and adsorption are recommended (Dwivedi et al. 2022).

**FIGURE 3:**
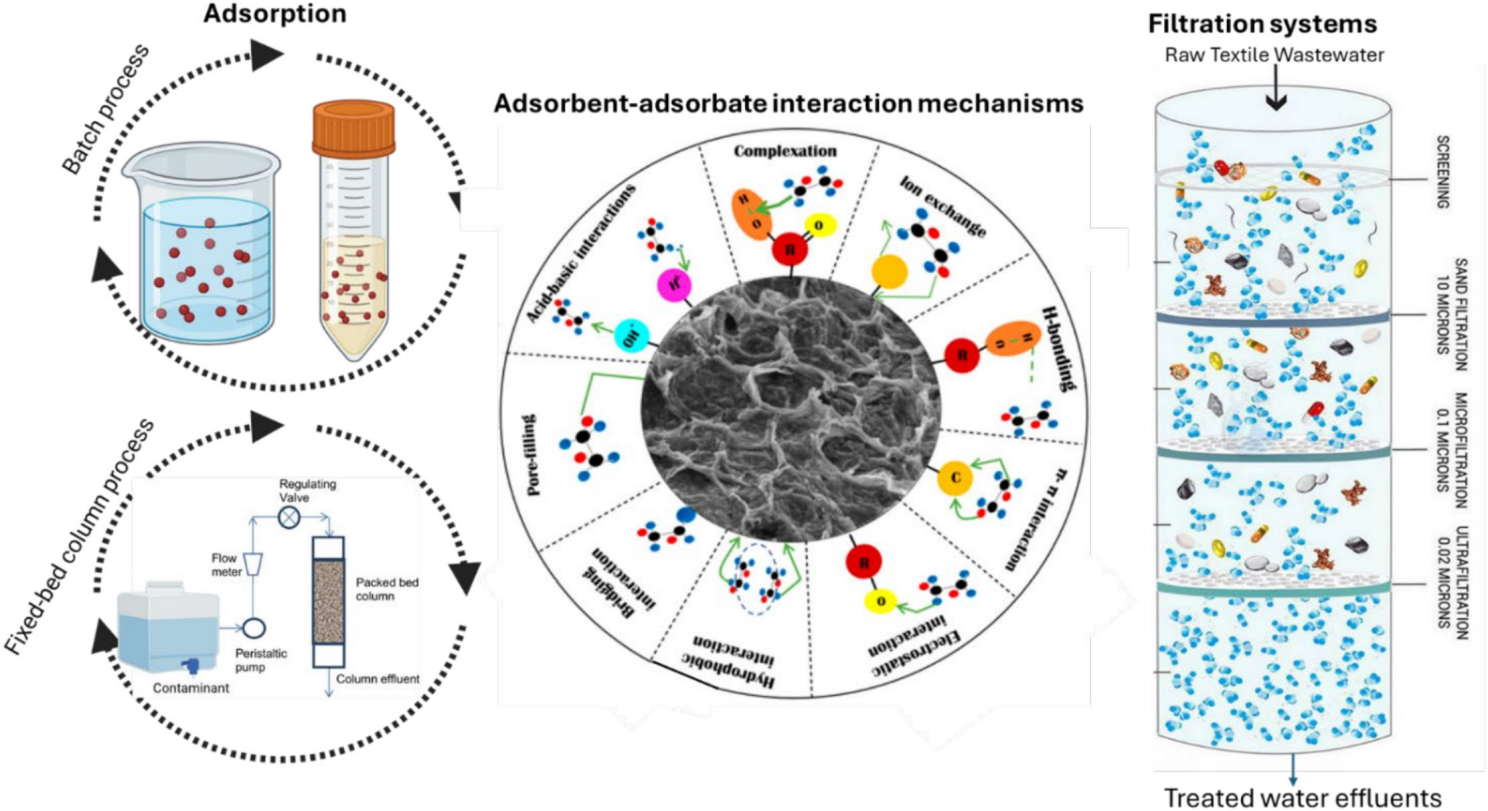
Representative schematic of the main physical treatment processes for textile wastewater, including adsorption and membrane filtration, as well as the dominant mechanisms responsible for contaminant removal in these systems.

Figure 3 illustrates the major physical treatment processes employed for textile wastewater remediation, namely adsorption and membrane filtration, together with the principal contaminant‐removal mechanisms associated with these technologies. The left panel depicts both batch and column adsorption systems, which represent the two most common operational modes used in laboratory investigations and practical treatment applications. Batch adsorption is widely used for preliminary screening of adsorbent performance and optimization of operating conditions, whereas column systems provide more realistic information on continuous‐flow treatment and scale‐up potential. The central panel summarizes the various adsorption mechanisms that govern contaminant removal, including electrostatic interactions, hydrogen bonding, hydrophobic interactions, π–π interactions, ion exchange, complexation, pore filling, and acid–base interactions. The relative contribution of these mechanisms depends on the physicochemical properties of both the adsorbent and the target contaminants, as well as operational parameters such as pH, ionic strength, and temperature. The right panel illustrates membrane‐based separation processes, highlighting the progressive removal of contaminants according to particle size through screening, sand filtration, microfiltration (MF), and UF. These membrane processes primarily operate through physical sieving and size exclusion mechanisms, enabling the removal of suspended solids, colloids, microorganisms, and selected dissolved pollutants.

### 5.1 Adsorption

Adsorption techniques have garnered considerable attention due to their high efficiency in removing a wide range of dyes (Adeola et al. 2023). The choice of an adsorbent depends on its affinity for the target contaminant, adsorption capacity, and regeneration ability (Adeola et al. 2022). While commercial activated carbon is highly effective for the removal of many contaminants due to its high surface area and porosity, its high cost due to energy‐intensive synthesis and regeneration challenges limits its widespread use in textile wastewater applications (Pet et al. 2024). To enhance the economic feasibility of adsorption treatment methods, researchers have explored low‐cost alternatives such as peat, bentonite clay, fly ash, polymeric resins, and biomass materials like wheat residue, treated ginger waste, groundnut shell charcoal, date stones, and potato plant waste (Sulyman et al. 2017). However, the application of these adsorbents is constrained by issues related to production scale‐up, regeneration, sludge production, and cost. A major challenge lies in the reduced efficiency of adsorbents when exposed to complex, multicomponent wastewater matrices where competitive adsorption can hinder selectivity and sorption capacity. Scale‐up from controlled experimental conditions to real‐world systems often reveals performance losses due to flow variability, fouling, and limited contact time (Satyam and Patra 2024).

#### 5.1.1 Experimental Approaches

Adsorption experiments are often conducted in the laboratory using either batch processes or fixed‐bed continuous flow systems, such as rapid small‐scale column tests (RSSCT), each offering distinct advantages and insights (Al‐Maas et al. 2022). Batch adsorption studies are employed for preliminary mechanistic investigations, providing data on adsorption capacity, equilibrium isotherms, and kinetics under controlled conditions (Adeola and Forbes 2019; Kubheka et al. 2022). Over 90% of adsorption studies reviewed in the current study included laboratory‐scale batch testing. In contrast, fixed‐bed column experiments using RSSCTs or similar flow‐through apparatus simulate real‐world continuous flow scenarios and are essential for scaling up adsorption systems for practical applications. RSSCT testing enables accelerated evaluation of important process design and operation factors, including contaminant breakthrough behavior, bed exhaustion time, and dynamic adsorption capacity (Matharage et al. 2025). Five of 12 adsorption studies reviewed in the current study included RSSCT or another form of flow‐through column testing.

#### 5.1.2 Adsorption Mechanisms

The removal of diverse contaminants from textile wastewater using carbon‐based nanomaterials and other natural/synthetic adsorbents involves a range of adsorption mechanisms that are governed by the physicochemical properties of both the adsorbates and the adsorbents (Ingrassia et al. 2023). Most prominent mechanisms include π–π interactions, especially for aromatic compounds like dyes, PAHs, and UV absorbers; electrostatic interactions and ion exchange, which are dominant in the sorption of charged species such as salts and heavy metals; while hydrogen bonding and hydrophobic interactions, which play critical roles in the removal of organic micropollutants including PFAS and phthalates (Figure 3). These adsorption processes are commonly described using isotherm models and equations (Figure S1). The Langmuir model, which assumes monolayer adsorption on a homogeneous surface; the Freundlich model, which accounts for heterogeneous surface energies; and the Sips model, a hybrid that captures both Langmuir and Freundlich behavior, especially at varying concentration ranges (Gujar et al. 2025). Kinetic models also provide insight into the adsorption pathways: pseudo‐first‐order kinetics are often associated with physisorption and ion exchange mechanisms, while pseudo‐second‐order kinetics are indicative of chemisorption processes such as hydrogen bonding or covalent interactions. Intraparticle diffusion models further elucidate the rate‐limiting steps, particularly for porous adsorbents, highlighting the significance of internal pore diffusion in the adsorption of larger or more hydrophobic molecules (Benjelloun et al. 2021). Overall, understanding the interplay between these mechanisms and models is critical for optimizing adsorption for multi‐contaminant removal in textile wastewater treatment. Ten of the 12 studies reviewed in the present study developed adsorption isotherms. Six of 10 reported that the Langmuir isotherm was the best fit to their data, 3 of 10 reported the Freundlich isotherm as the best fit model, and 1 of 10 reported a different isotherm.

**FIGURE 4:**
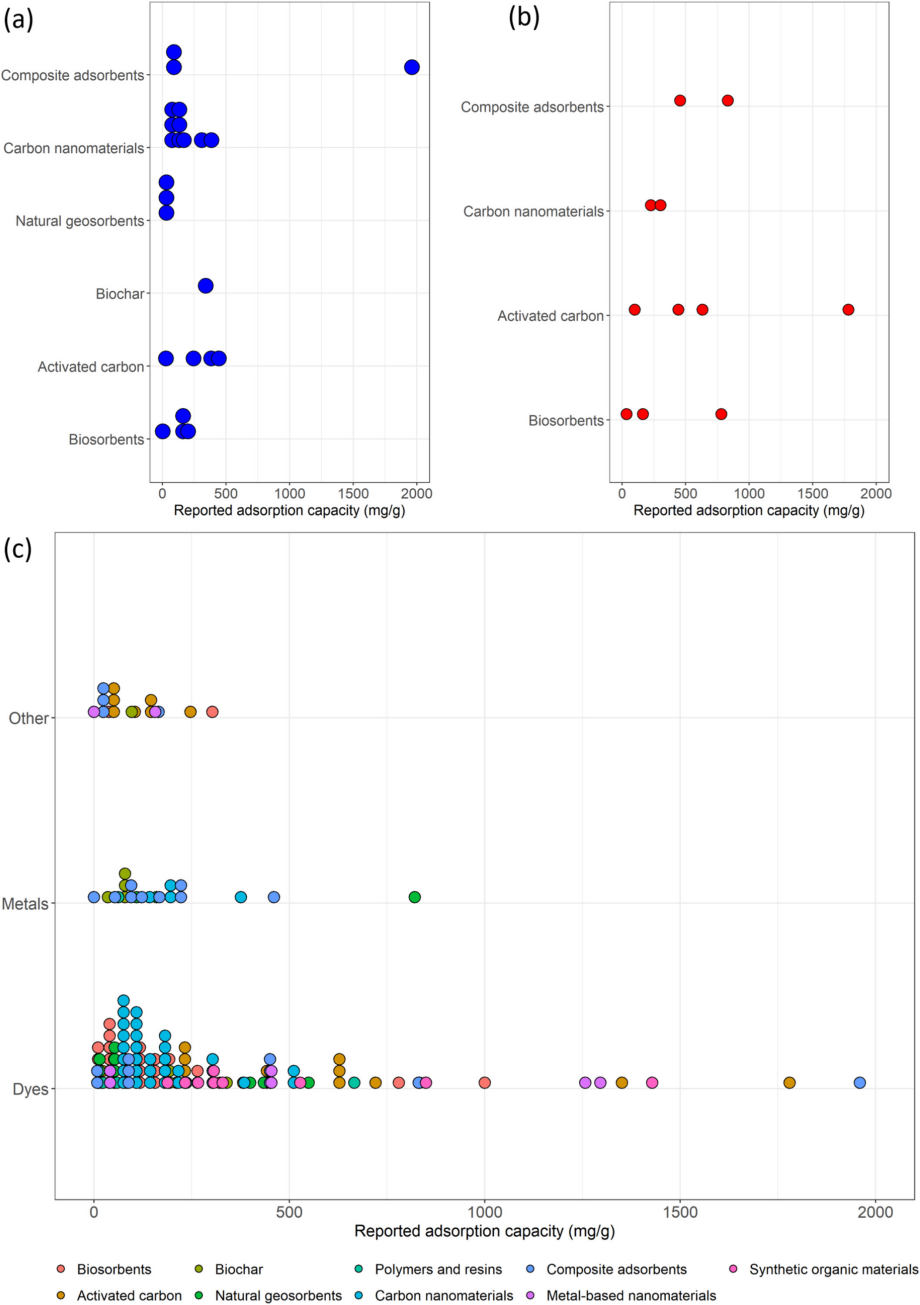
Reported laboratory performance of various adsorbents for the removal of contaminants present in textile wastewater: (a) methylene blue, (b) Congo red, and (c) dyes, metals, and other contaminants (adapted from data reported in Adeola et al. 2022; Afshari and Dinari 2020; Benjelloun et al. 2021; Gunday et al. 2025; Ighalo et al. 2022; Ingrassia et al. 2023; Jejurkar et al. 2020; Khalilzadeh Shirazi et al. 2020; Mokhtari et al. 2020; Wang, Li, et al. 2022).

On a practical level, the goal of isotherm experiments is to establish the adsorption capacity of novel adsorbents under relevant conditions (influent concentration of contaminant, temperature, pH, etc.) so that they can be compared to one another and to more established and widely used adsorbents like activated carbon. Figure 4 summarizes results published in previous review studies and research papers (Ighalo et al. 2022; Adeola et al. 2022; Ingrassia et al. 2023; Benjelloun et al. 2021), which focused predominantly on carbon‐based adsorbents and targeted dye‐based contaminants or metals. Although the data in Figure 4 does not encompass all past research on the topic of adsorption for contaminant removal in the context of textile wastewater treatment and does not account for differences in experimental conditions between studies. It nonetheless demonstrates the wide range of adsorption capacities that have been reported in past studies and that many of the novel adsorbents developed in past studies have low or moderate adsorption capacities relative to activated carbon. Figure 4a,b reflects that the adsorption capacity for prominent representative dyes such as methylene blue (MB) and Congo red (CR) mostly falls below 500 mg/g, CR showing better sorption affinity with AC and biosorbents than MB. The tight clustering near 500 mg/g adsorption capacity provides a starting point for engineering studies, as these synthetic and natural sorbents mostly remove metals, dyes, and other contaminants around that maximum capacity (Figure 4c). The results also emphasize the need for consistent experimental procedures and reporting across studies to facilitate meaningful comparison of materials and the identification of promising classes of materials.

Kinetic studies, which elucidate the rate and duration of interaction between the adsorbate and the adsorbent, provide critical insights for the design and optimization of full‐scale adsorption systems. Eight of the 12 adsorption studies reviewed for this paper included detailed kinetic investigations.

#### 5.1.3 Technoeconomic Analysis of Adsorption Treatment Processes

Only limited information about the design, operation, and costing of full‐scale textile wastewater processes, including adsorption‐based processes, is available in the public sphere or the peer‐reviewed literature. This makes it difficult to compare the feasibility and cost‐effectiveness of the diverse adsorbents that have been developed for textile wastewater remediation at the laboratory scale. The US Environmental Protection Agency (USEPA 2021) has developed a costing model for adsorption‐based water treatment processes that incorporates:

Capital costs for treatment equipment (design and average flow rate, number and size of contactors, initial adsorbent supply, etc.)
Capital costs for other equipment (pumps, instrumentation, chemical addition, etc.)
Capital costs for building
Indirect and add‐on capital costs (system design work, site work, construction management, equipment transportation and installation, contractor fees, permitting, pilot studies, financing, etc.)
Operating costs related to adsorbent (adsorbent regeneration, replacement, residual discharge fees)
Other operating costs (labor, electricity, other chemicals, maintenance on equipment and buildings)

- Cost to synthesize a unit of adsorbent.

Although most of these cost items are site‐specific and beyond the scope of laboratory‐based studies, the characteristics of the adsorbent will impact multiple cost categories. For example, the affinity of the adsorbent for the target contaminants will affect the size and number of contactors required (capital cost of treatment equipment), the need for pH adjustment or other water quality changes to optimize treatment (capital and operating costs of other equipment), and the adsorbent regeneration or replacement frequency (operating costs related to adsorbent). Other costs are site‐specific (e.g., site preparation), jurisdiction‐specific (e.g., labor), or time‐specific (e.g., electrical costs). In the absence of design, operation, and costing information for existing textile wastewater installations, past studies have attempted to quantify, or at least compare on a semi‐quantitative basis, (a) the cost of synthesizing adsorbents and (b) the cost to remove a unit amount of adsorbate using the adsorbent.

A key metric for comparative techno‐economic analysis of adsorption materials is the cost to synthesize one unit mass of adsorbent (Ighalo et al. 2022). Among chemically modified biopolymers, succinylated chitosan represents a high‐performance but relatively expensive option (Gkika et al. 2019). This material is produced by grafting succinic anhydride onto chitosan's amino groups under mild stirring and ambient‐to‐moderate temperatures, followed by multi‐step purification involving solvent precipitation, dialysis, and drying (Kyzas et al. 2015). The cost drivers/components for this route include high‐purity reagents (succinic anhydride, acetic acid, methanol/ethanol), energy for drying, and labor‐intensive processing. Consequently, the synthesis cost of such specialized polymeric adsorbents can exceed US$20,000 kg^−1^ (Gkika et al. 2019), reflecting the trade‐off between enhanced adsorption performance and economic scalability.

In contrast, bio‐ and agro‐waste‐derived adsorbents can offer a more cost‐effective alternative. For example, it has been reported that rice‐hull and garlic‐stalk biochars have an estimated preparation cost of US$1.08 kg^−1^, which can drop to US$0.11 kg^−1^ when natural drying is employed (Yetgin and Amlani 2024). Similarly, acid‐activated carbon prepared from sawdust incurs a higher cost of US$8.34 kg^−1^ due to energy‐intensive furnace use, phosphoric acid consumption, and acid‐washing steps (Shah et al. 2020).

Hydrothermal and solvothermal syntheses are widely used to prepare advanced metal oxide and carbon‐based adsorbents, and these methods require sealed, high‐pressure reactors, elevated temperatures (often 120°C–250°C), and sometimes costly organic solvents, which elevate operational and capital costs (Nadimpalli et al. 2018; Zhang, Biswal, et al. 2023). These requirements typically lead to production costs with further scale‐up challenged by reactor design and energy recovery. In contrast, simpler thermal methods such as pyrolysis and carbonization, especially when applied to low‐cost biomass feedstocks (e.g., agricultural residues) in simpler equipment, result in significantly lower costs (Pahnila et al. 2023). On the other hand, polymerization techniques drive costs higher due to the use of high‐purity monomers, crosslinkers, initiators, and solvent systems, along with multiple reaction and purification steps (Reis et al. 2020). Production costs for specialized polymeric adsorbents can reach US$6000–22,000 per kg, making them viable predominantly for specialized applications rather than bulk wastewater treatment (Gkika et al. 2019). Meanwhile, physical methods such as laser ablation, plasma synthesis, or arc‐discharge for nanoparticle production carry the highest cost because of high energy consumption, low yields and high equipment investment, making them unsuitable for large‐scale adsorption applications despite their superior material control.

The cost‐effectiveness of adsorbent manufacturing is best maximized when low‐cost feedstocks (e.g., biomass wastes) are used with mild processing requirements (e.g., low‐temperature pyrolysis) rather than high‐pressure/temperature synthetic methods. Moreover, future techno‐economic studies should adopt standardized metrics and provide detailed breakdowns of costs for raw materials, energy requirements, labor, and waste disposal requirements. These enable realistic comparisons between synthesis routes and support informed material selection, particularly when scaling from lab to industrial wastewater‐treatment systems.

2 Cost to remove a unit amount of adsorbate using the adsorbent.

While adsorption capacity is commonly reported as a measure of performance, it does not account for the costs associated with adsorbent production and operation. Therefore, cost‐normalized metrics are necessary to facilitate meaningful comparisons among different adsorbent materials. One such metric is the adsorption cost, expressed as the cost required to remove a unit mass of contaminant (US$ mol^−1^ contaminant removed) (Bajić et al. 2016). To improve comparability across studies, all costs discussed in this review were adjusted to their January 2026 equivalent values using the CPI, thereby accounting for inflation‐related changes in purchasing power and allowing more consistent economic comparisons among studies conducted in different years.

Studies suggest that sorbent costs can vary widely, with most falling in the range of US$1.10 to US$219.61 mol^−1^ (Ighalo et al. 2022). Generally, sorbents priced below US$1.10 mol^−1^ are considered cheap, offering promising economic viability, while those exceeding US$219.61 mol^−1^ are regarded as expensive applications. Collectively, recent research emphasizes that bio‐based and waste‐derived materials may offer cheaper alternatives compared with conventional adsorbents. For instance, a cost analysis of tomato seed‐based sorbent for dye removal revealed a competitive cost of US$1.56 mol^−1^ for removing 1 g of dye, while activated carbon derived from gasification char residues achieved an even lower cost of US$0.25 mol^−1^, significantly cheaper than commercial activated carbon, which averages around US$4.98 mol^−1^ (based on Sigma‐Aldrich pricing) (Ahmad et al. 2023; Ahmad et al. 2020). Similarly, biochars produced from agricultural residues such as groundnut shells, rice husks, and coconut shells have shown cost advantages, with groundnut shell biochar achieving the lowest dye removal cost at US$160 kg^−1^ compared with US$180 and US$820 kg^−1^ for rice husk and coconut shell biochars, respectively. These findings collectively indicate that agro‐industrial wastes can serve as sustainable, cost‐effective sorbents while valorizing otherwise discarded biomass.

Additional studies extend these observations to other unconventional sorbents and contaminants. Nikolić et al. (2020) investigated the removal of Cu (II) ions from aqueous solutions using electric arc furnace slag and alkali‐activated slag, reporting treatment costs of US$0.03 and US$0.1 kg^−1^, respectively, with the higher cost of alkali‐activated slag attributed primarily to the preparation of the alkali activator (Nikolić et al. 2020). Similarly, Pap et al. (2020) evaluated crab carapace–based adsorbents for phosphorus recovery from wastewater, achieving a treatment cost of US$264.6 kg^−1^, significantly lower than conventional technologies such as activated carbon, RO, ion exchange, or electrolysis (Pap et al. 2020). The reduced cost was partly attributed to minimal biofouling observed with calcite–chitosan‐based adsorbents. Overall, these studies demonstrate that sorbents derived from agro‐waste, industrial byproducts, and other unconventional sources offer economically viable alternatives to traditional adsorbents, providing both cost savings and sustainability benefits for diverse water treatment applications.

### 5.2 Membrane Filtration

Membrane‐based technologies are used in full scale textile wastewater treatment installations (see Section 3) and have gained significant research attention for textile wastewater treatment due to their ability to remove dyes, suspended solids, dissolved salts, and organic contaminants while enabling water reuse and resource recovery (Obotey Ezugbe and Rathilal 2020). However, these processes differ substantially in their separation mechanisms, contaminant targets, and operational requirements. MF and UF are low‐pressure membrane processes primarily used for the removal of suspended solids, colloidal particles, microorganisms, and high‐molecular‐weight organic compounds through size exclusion (Bardhan et al. 2022). As such, they are commonly employed as pretreatment steps to protect downstream treatment units. NF operates at higher pressures and can effectively remove multivalent ions, color, dyes, and dissolved organic compounds through a combination of size exclusion and charge‐based (Donnan) interactions (Namla et al. 2025). Numerous studies have demonstrated that NF membranes are among the most effective separation methods for treating textile dyeing effluents (Yi et al. 2025). RO provides the highest level of separation among pressure‐driven membrane technologies and can reject most dissolved salts, organic contaminants, and micropollutants, thereby producing high‐quality water suitable for reuse (Liu, Livingston, et al. 2024).

Emerging membrane technologies such as membrane distillation (MD) and electrodialysis (ED) have also shown promise for textile wastewater treatment. MD utilizes a hydrophobic membrane and a temperature gradient to transport water vapor while retaining dissolved contaminants, making it particularly attractive for treating high‐salinity wastewater (Sheng et al. 2025). ED, on the other hand, employs ion‐exchange membranes and an electric field to selectively separate dissolved ions, enabling salt recovery and wastewater desalination with lower hydraulic pressure requirements than RO (Gurreri et al. 2020).

In addition to size exclusion and pore‐based separation mechanisms, membrane surfaces may engage in hydrogen bonding, covalent interactions, and electrostatic attraction or repulsion with various contaminants in textile wastewater (Lu et al. 2022). For instance, membranes functionalized with oxygen‐ or nitrogen‐containing groups can form hydrogen bonds with polar contaminants such as dyes or PFAS (Xu et al. 2025). Similarly, charged membrane surfaces can facilitate electrostatic interactions with ionic species such as salts and heavy metals, enhancing removal efficiency beyond sieving (Beshahwored et al. 2024). In some advanced membrane systems incorporating reactive or functional nanomaterials (e.g., graphene oxide, metal–organic frameworks), covalent interactions or π–π stacking may also occur, particularly with aromatic or electron‐rich organic compounds such as PAHs and UV absorbers (Fu and Gray 2024). Depending on the material and surface chemistry of membranes, adsorption‐based interactions can also play a significant role in contaminant removal. The traditional filtration theory often overlooks the possibility of adsorption‐driven processes that may occur concurrently, which contribute to hybrid separation effects and improve the selectivity and retention performance of membrane‐based water treatment processes (Al‐Ghouti et al. 2023).

Although membranes are used in some full‐scale textile wastewater treatment installations, currently, most research remains focused on laboratory or pilot‐scale applications. Therefore, extensive investigations into the long‐term performance of NF membranes are essential to understanding how specific components in textile wastewater contribute to membrane fouling. Such insights could facilitate the development of effective pretreatment systems, potentially via permeability enhancement and reduction of fouling during long‐term operations.

Despite their advantages, membrane processes face several operational challenges, including membrane fouling, scaling, concentrate management, and material degradation. To achieve lower capital costs and higher productivity, integrated membrane systems combining conventional and membrane‐based treatments have been widely explored (Rezende Moreira et al. 2022). However, membrane fouling is a significant challenge, particularly due to the complexity of textile wastewater, and the role of dyes and salts in the formation of colloidal fouling layers on membrane surfaces (Egabaierdi et al. 2022).

#### 5.2.1 Technoeconomic Assessment of Membrane Filtration for Textile Wastewater Treatment

Publicly available data on the techno‐economic performance of membrane filtration systems for textile wastewater treatment remain limited, largely due to variations in scale, influent composition, and membrane configurations used across studies. Ciardelli et al. (2001) estimated that treating dyehouse effluents using membrane‐based techniques in Italy would cost approximately US$2.40 m^−3^ (adjusted to 2025 values), with anticipated increases linked to the global escalation in chemical and energy costs. Similarly, studies from India reported treatment and recovery costs of around US$1.80 m^−3^ for integrated RO and NF systems, costs that are substantial, but remain lower than the price of purchasing high‐quality freshwater for textile dyeing operations in water‐stressed industrial regions such as Tirupur (Ranganathan et al. 2007).

In contrast, Ersahin et al. (2018) documented a significantly lower treatment cost of US$0.45 m^−3^ in Istanbul, Turkey, using a pressure‐driven hybrid membrane system combining sand filtration, UF, and RO units. Earlier studies by Marcucci et al. (2001) in Italy reported comparable performance, with water treatment costs estimated at approximately US$0.45 m^−3^ using a multi‐barrier system comprising UF and sand filtration (Marcucci et al. 2003; Marcucci et al. 2001). Likewise, Madwar and Tarazi (2003) evaluated a wastewater desalination plant incorporating RO and found recovery costs of US$0.47 (Madwar and Tarazi 2003), while Defrawy (2002) reported slightly lower costs of US$0.43 m^−3^ for UF–RO combinations designed for textile wastewater. Across these studies, the primary cost components included capital investment, energy consumption, membrane replacement, and labor (Defrawy 2002). Operational costs depend heavily on influent wastewater. Future techno‐economic assessments should therefore integrate life‐cycle costing and resource recovery metrics to ensure the sustainable implementation of membrane filtration technologies in the textile sector.

### 5.3 Summary of Physical Treatment Processes for Textile Wastewater Treatment

Table 1 highlights that adsorption and membrane‐based processes remain the most extensively investigated physical treatment technologies for textile wastewater remediation. Reported removal efficiencies are generally high, ranging from approximately 55%–100% for color, dyes, COD, salts, and selected heavy metals, depending on wastewater composition and operating conditions. Adsorption studies predominantly target dyes and organic pollutants, while membrane processes such as UF, NF, ED, and hybrid systems demonstrate strong performance for simultaneous dye, salt, and COD removal, supporting water reuse and resource recovery objectives. Notably, the majority of reported studies were conducted at laboratory scale, with relatively few semi‐pilot and pilot‐scale investigations. This highlights a significant gap between laboratory performance and industrial implementation, emphasizing the need for long‐term pilot‐scale validation, economic assessment, membrane fouling studies, and evaluation under real textile wastewater conditions before broader commercial adoption can be achieved.

**TABLE 1:** Physical processes and performance metrics in textile wastewater treatment.

Physical processes	Target compounds	Optimal conditions	Mechanisms, removal efficiency	Research scale	References
Adsorption using lignin‐based adsorbents	Rhodamine B	3 g/L sorbent dose, 720 min contact time and under acidic pH conditions	Sips, D‐R and intraparticle diffusion models/∼97%	Lab scale	(Adeola et al. 2023)
Adsorption using Palm shell‐AC	Methylene blue	0.1 mg/250 mL, pH ~6.5 at 30°C	Langmuir, Pseudo‐second order (PSO/sorption capacity: 243.90 mg/g)	Lab scale	(Tan et al. 2008)
Adsorption using *Rumex abyssinicus* plant‐derived activated carbon	Methylene blue from textile industrial wastewater	Sorbent dosage of 60 mg/100 mL, contact time of 60 min, pH 9.	Freundlich, Pseudo‐second order (PSO)/∼100%	Semi‐pilot	(Fito et al. 2023)
Adsorption using natural materials (geosorbents)	Chromium, copper, cadmium, zinc, COD in textile effluents	2 g/L dosage of adsorbent, 160 min contact time, pH 10.	Langmuir, PSO/Cr (∼24%–38%), Cu (∼19%–28%), Cd (∼19%–36%), Zn (∼24%–40%), COD (∼80%–89%)	Lab scale	(Assila et al. 2020)
Adsorption onto coal fly ash	Color, COD	Dose of 12–40 g/L, with an initial agitation step of 3–5 min, pH ≤ 2 with a coal ash	∼55%–83% Color, ∼44%–61% COD	Lab scale	(Zaharia and Suteu 2013)
Graphene oxide	COD	Adsorbent dose: 0.125 g/L, duration: 25 min, pH 8.	∼62% COD	Semi‐pilot	(Maroufi et al. 2021)
Sand filtration, UF and NF (DL 4040F Osmonics)	COD, total suspended solids (TSS), dye	COD 142 mg/L, TSS 12 mg/L, conductivity 3950 μS/cm, pressure of 0.4 and 9 bars, pH 7.8	NA/ > 93% COD, > 60% COD	Pilot scale	(Marcucci et al. 2001)
NF with Desal 5 DK (Osmonics)	Dye	Pressure of 10 bar, temperature 25°C and pH 6 with and without stirring	NA/100% Direct Red 80	Semi‐pilot	(Akbari et al. 2002)
Polyamide‐based NF membrane	Dyes	1 g/L dye, followed by 2.5 g/L of NaOH, 1 g/L of Na_2_SO_3_, 0.2 g/L of EDTA, 11 and 19 g/L of Na_2_SO_4_.	Freundlich/99 and > 99.3% for Reactive Blue 2 and Reactive Orange 16	Lab scale	(Van der Bruggen et al. 2001)
NF membrane in film‐thin composite	Dyes, COD	120 min of the operation time, sodium chloride added, 10–12 bar pressure	98% dyes, 100% COD	Lab scale	(Hassani et al. 2008)
Flat sheet NF membrane (NTR 7450‐ Nitto–Denko)	Salts and dyes	Pressures of 0–60 bar, crossflow velocity of 0–0.75 m/s, Reactive Orange 16 or Reactive Blue 2 (15 g/L), Na_2_SO_4_ (56 g/L), surfactant‐EDTA (0.2 g/L), Na_2_SO_3_(1 g/L) and NaOH (2.5 g/L).	∼92% dye removal and ∼87% salt removal	Lab scale	(Hassani et al. 2008)
Hybrid Tight UF/Bipolar‐Membrane Electrodialysis (TUF/BMED)	Na_2_SO_4_, dyes	TUF pre‐treatment; BMED with external pH control	Na_2_SO_4_ removal: > 90%; Dye loss: < 5%	Lab scale	(Lin et al. 2019)
Hybrid UF/Electrodialysis (UF/ED)	COD, salts, color	UF with ceramic membrane; ED at 25 min	TDS removal: 94.2%; Conductivity removal: 97.1%	Lab scale	(Lafi et al. 2018)
NF‐based electrodialysis	Dyes, salts	Current of 0.5 A, power voltage range of 60.4 V, temperature of 25°C, conductivity of the feed of 1.0 mS/cm, feedstock of 1.0 g/L Reactive Orange and 12.0 g/L NaCl	∼99% salt removal efficiency and ∼99% dye recovery	Semi‐pilot	(Ye et al. 2020)

*Note:* Lab scale: conducted in a controlled laboratory environment using small quantities of materials/volume of solutions. Semi‐pilot scale: intermediate testing with partial process integration, larger than lab scale. Pilot scale: Pre‐commercial testing system simulating real conditions to validate scalability. Full scale: Complete, operational system implemented for commercial or industrial use.

## 6 Chemical Processes for the Treatment of Textile Wastewater

**FIGURE 5:**
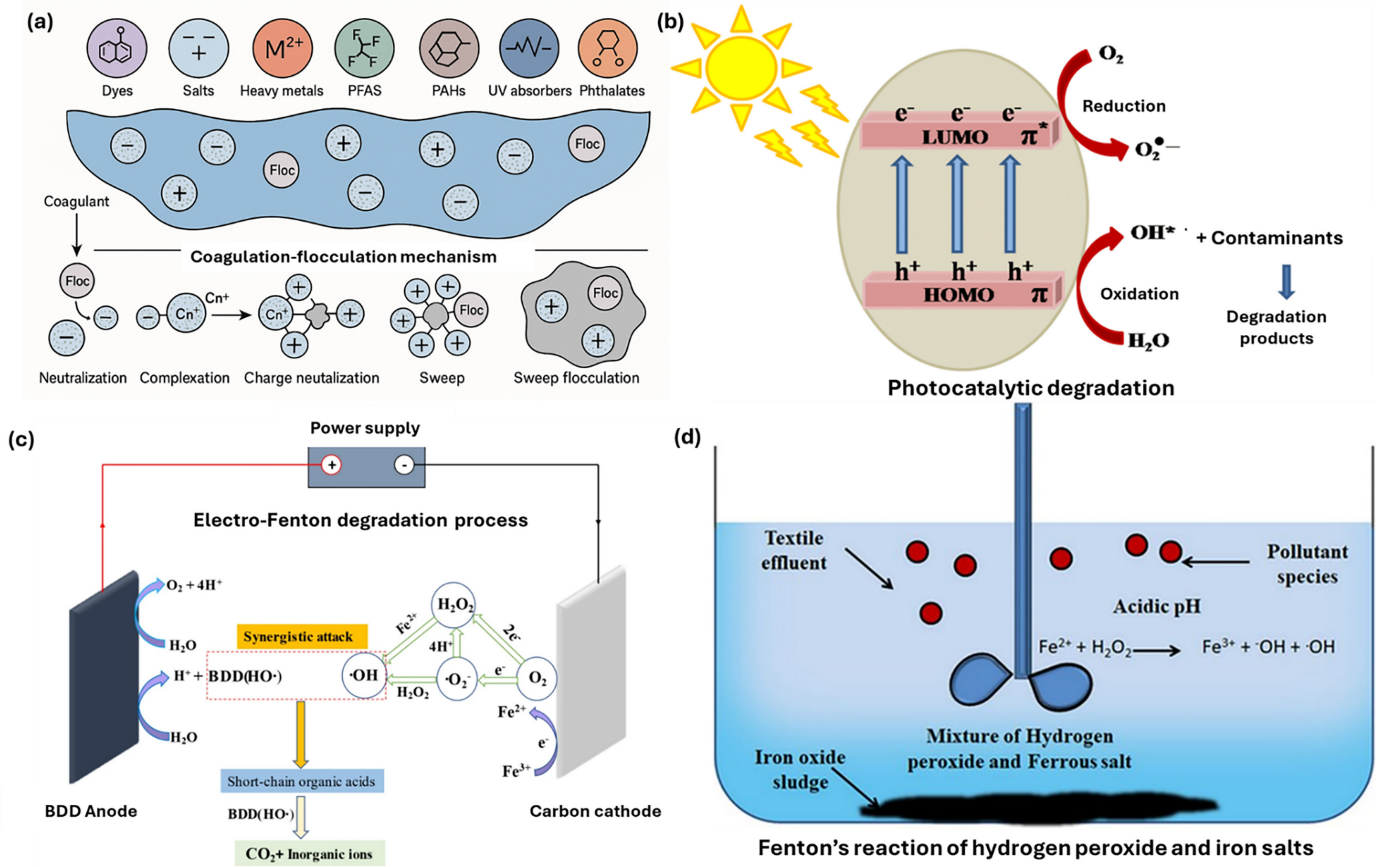
Mechanisms of chemical treatment processes for textile wastewater treatment reported in previous studies (a) coagulation‐flocculation (b) UV‐driven photocatalytic degradation (c) electro‐Fenton degradation/electrochemical degradation (d) Fenton process with H_2_0_2_ and iron salt. Adapted with modifications from (Kallawar and Bhanvase 2024; Saha et al. 2020).

Chemical treatment processes use chemical agents to facilitate the degradation or removal of toxicants, and these chemical agents can either be introduced externally or produced in situ (Tenzin et al. 2022). Table 2 demonstrates that chemical treatment technologies, including coagulation–flocculation, ozonation, AOPs, electrochemical treatment, and hybrid systems, are highly effective for the removal of color, dyes, COD, suspended solids, salts, and selected CECs from textile wastewater. Reported treatment efficiencies range from 35% to nearly 100%, with photocatalytic and electrochemical processes often achieving the highest removal rates for recalcitrant organic pollutants and dyes. Conventional coagulation–flocculation remains attractive due to its operational simplicity and widespread uptake for municipal water and wastewater treatment, whereas advanced oxidation and electrochemical technologies offer superior degradation of persistent compounds. Hybrid treatment systems frequently outperform standalone processes by combining complementary removal mechanisms, such as those captured in Figure 5. However, most studies remain at the laboratory scale, with only a limited number of semi‐pilot and pilot‐scale investigations reported. This highlights the need for further scale‐up studies, long‐term operational assessments, energy and chemical consumption analyses, and techno‐economic evaluations under real textile wastewater conditions to support wider industrial implementation.

**TABLE 2:** Chemical processes and performance metrics in textile wastewater treatment.

Chemical processes	Target compounds	Optimal conditions	Efficiency (%)	Research scale	References
Coagulation–flocculation	COD, TSS, color, chromium	pH 7; 600 ppm alum; 1.5 ppm polyacrylamide; 240 rpm (1 min), 40 rpm (20 min); 30 min settling	COD: 58.55%; TSS: 65%; Color: 78.96%; Chromium: 76.45%	Lab scale	(El‐Gohary and Tawfik 2009)
Coagulation–flocculation	COD, TSS, color	pH 7.5; 800 ppm PAC; 1 ppm polyacrylamide	COD: 65.4%; TSS: 67.5%; Color: 75.49%	Lab scale	(Verma et al. 2012)
Coagulation–flocculation	Textile fibers	pH ~7; 0.72–3.94 mM polyaluminum chloride (PACl)/FeCl_3_	57%–91% removal with PACl; 67%–99% with FeCl_3_;	Lab scale	(Vasiljević et al. 2023)
Coagulation‐flocculation	Turbidity, COD, color, TSS	*Azadirachta indica A. Juss*/ *Moringa oleifera* extract, pH 7–8; dosage 50–200 mg/L; rapid mixing 1–3 min, slow mixing 15–20 min; 30 min settling	Turbidity: 85%–95%; Color: 70%–80%; TSS: 65%–75%, COD: 98.5% *Escherichia coli* : 100%	Lab scale	(Hoa and Hue 2018; Thirugnanasambandham and Karri 2021)
Ozonation	COD, dye	Flow rate 0.042 m^3^/h, 10 min contact time, stirring at 250 rpm	80% Color, 65% COD	Lab scale	(Basak SaO 2015)
Coagulation with ozonation	Color, COD	Ozone dosage: 675 mg/L; FeCl_3_ dosage: 800 ppm; pH: 5	Color: 83%; COD: 96.5%	Lab scale	(Malik et al. 2017)
Biological aerated filters + coagulation/flocculation	COD, TOC, BOD_5_, color, nutrients (N, P), toxicity	—	BOD_5_ removal 98%; COD ≈87%; TOC ≈ 88%; color > 81%; total phosphorus ~98%; toxicity ( *Vibrio fischeri* ) ~98% reduction; total nitrogen ~48% removal	Lab scale	(Paździor et al. 2017)
Bipolar membrane electrodialysis	Salts, conductivity	pH 8; 15 V; 60 min	Conductivity reduction: 85%	Lab scale	(Yuzer and Selcuk 2021)
Electrochemical mineralization using boron‐doped diamond	COD in textile effluents	60 mA/cm^2^ current density, 3 h, 3.0 g/L NaCl, pH 2.0	∼100%	Semi‐pilot	(Zou et al. 2017)
Electrolytic degradation	Azo dyes, BOD, COD in textile wastewater	12 V potential, current of 130 A, flow rate of 6 dm^3^/min, r 0.02 M NaCl electrolyte	94.4% dyes; 35% and 45% reduction in COD and BOD	Pilot	(Sakalis et al. 2005)
UV‐driven degradation using Glucose‐tailored SnO_2_/TiO_2_/RGO	Rhodamine B Methylene blue	500 W mercury lamp (λ < 420 nm), dosage of 10 mg/100 mL, 0.667 h	97.6% 99.5%	Lab scale	(Jiang et al. 2022)
Sunlight‐driven degradation using Co‐SnO_2_/SGCN	Methylene blue	Irradiation of sunlight (68–73 klux), catalyst dosage of 0.2 g/100 mL, 2.5 h	96.0%	Lab scale	(Javed et al. 2023)
Xenon lamp‐derived degradation using SnO_2_/TiO_2_	Rhodamine B Methyl orange	Catalyst dosage 20 mg/20 mL, current density (0.55 μA/cm^2^), 300 W xenon lamp, 2 h	93.0% 91.0%	Lab scale	(Huang et al. 2022)
Solar light‐mediated degradation with SnO_2_/MoS_2_/rGO	Methylene blue	1.25 h, 20 mg catalyst	90.0%	Lab scale	(Agboola and Shakir 2022)
Electro‐oxidation using PbO_2_–Ti anode/Ti mesh‐plate cathode	COD in printing and dyeing wastewater	Current density of 10 mA/cm, pH: 8.3, 40 min, 12.04 kWh/m^3^	72% COD	Lab scale	(Wang et al. 2018)
Sunlight‐driven degradation using BiVO_4_/Bi2S_3_/MoS_2_	Rhodamine B, methylene blue, and malachite green	Catalyst dosage of 0.1 g, 5 h, scavenger of 2 mM isopropanol	97%, 93%, and 94%	Lab scale	(Wang et al. 2017)
Persulfate‐mediated solar photo‐Fenton (solar/Fe/S_2_O_8_^2−^)	CECs: caffeine, carbendazim, losartan; *E. coli* ; antibiotic‐resistant bacteria (ARB)	Neutral pH; intermittent Fe^2+^ additions; accumulated irradiation ~1.9 Kj/L; Fe^2+^ ca. 27.7 mg/L; persulfate 0.3–1.5 mM	CEC removal ~55%; *E. coli* ~3 log units; ARB ~3–4 log units	Semi‐pilot scale	(Mc et al. 2021)
Catalytic ozonation after electrocoagulation, AC catalyst	Reactive Black 5 dye wastewater; colorless by‐products; COD, TOC	Real industrial wastewater; polishing step after EC; using activated carbon (AC) catalyst; ozonation duration (not fully specified)	Color removal (to colorless); COD removal ~40%; TOC removal ~35% when using AC; much lower COD/TOC removal without AC (≈18%–23%)	Lab scale	(Bilińska et al. 2016)

Abbreviations: BOD: biochemical oxygen demand; COD: chemical oxygen demand; TOC: total organic carbon; TSS: total suspended solids.

### 6.1 Coagulation and Flocculation

Coagulation and flocculation processes involve the addition of a coagulant like aluminum sulfate that generates “flocs” that interact with water contaminants, making them easier to remove via downstream sedimentation and filtration processes (Figure 5a). Coagulants work by neutralizing particle charges to destabilize them and facilitate settling, whereas flocculants promote the aggregation of these particles into larger, settleable clusters (Badawi et al. 2023). Coagulation–precipitation is another process that has been associated with the treatment of wastewater, and it involves forming insoluble salts from dissolved ions, while coagulation–flocculation involves destabilizing and aggregating suspended particles into larger, settleable flocs (Pillai and Thombre 2020).

Coagulation‐based methods are effective for decolorizing textile wastewater containing dispersed dyes, but this method is less efficient when the wastewater contains reactive and vat dyes (Verma et al. 2012). The coagulation–flocculation process is not highly effective for removing reactive dyes. Even with the addition of a flocculant, the poor quality of the floc may lead to uneven settling (Kono 2017). This is because reactive dyes are highly water‐soluble and form strong chemical bonds with water molecules, making them harder to destabilize and aggregate. Their molecular structure typically includes ionic groups (like sulfonate groups), which increase their electrostatic repulsion and resistance to charge neutralization during coagulation (Lau and Ismail 2009).

A major limitation of coagulation‐based treatment is the generation of large volumes of sludge, which increases handling, dewatering, transportation, and disposal costs (Golob et al. 2005; Liang et al. 2014).

### 6.2 Ozonation and Ozone Advanced Oxidation Processes (O_3_‐AOPs)

Ozonation involves the application of ozone (O_3_), a powerful oxidizing and disinfecting agent, to degrade organic contaminants and improve wastewater quality (Lim et al. 2022). In direct ozonation, ozone reacts selectively with electron‐rich compounds such as dyes, chromophores, and certain organic pollutants, breaking them into smaller and often more biodegradable molecules (Sadek et al. 2026). Ozonation has been identified as an effective pre‐treatment and post‐treatment technology for textile wastewater due to its ability to reduce color, COD, and toxicity (Radetski et al. 2002). A typical ozonation system consists of an ozone generator, an oxygen or air supply unit, a contactor or reaction tank, and an ozone destruction unit to safely remove residual ozone (Jekel 2000; Wei et al. 2017).

In contrast, ozone‐based AOPs combine ozone with additional agents such as hydrogen peroxide (O_3_/H_2_O_2_), ultraviolet radiation (O_3_/UV), catalysts, or photocatalysts to generate highly reactive hydroxyl radicals (˙OH) (Wang et al. 2026). These radicals are less selective and possess higher oxidation potentials than molecular ozone, enabling the degradation of a broader range of persistent and recalcitrant contaminants. In recent years, research and industrial applications have increasingly focused on ozone‐based AOPs rather than standalone ozonation, owing to the enhanced generation of hydroxyl radicals and improved degradation of recalcitrant pollutants.

A comprehensive evaluation of ozone‐based AOPs for textile wastewater treatment by Bilińska et al. (2016) compared simulated dye solutions and real industrial effluents containing Reactive Black 5 and auxiliary chemicals. The study assessed O_3_, O_3_/H_2_O_2_ (peroxone), UV/O_3_, and O_3_/UV/H_2_O_2_ systems, achieving nearly complete color removal (≈100%) across all ozone‐based processes. However, treatment efficiency depended strongly on wastewater composition, with COD and TOC reductions reaching approximately 90% and 50% in synthetic dye solutions but only approximately 10% and 20% in real effluents, reflecting the inhibitory effects of complex organic additives and salts (Bilińska et al. 2016). Ozonation improved biodegradability, increasing the BOD_5_/COD ratio from 0.02 to 0.30 and enhancing the average oxidation state of organic matter, while *Vibrio fischeri* assays showed a marked toxicity reduction (EC_50_ rising from 3.86 ± 0.32 mg/L to > 100 mg/L). Estimated ozone consumption was 0.05–0.1 kg O_3_ m^−3^, with O_3_ and O_3_/H_2_O_2_ (0.005 M) identified as the most cost‐effective options. Exposure to UV radiation slightly improved oxidation but also increased operational costs.

In another practical application, textile wastewater with an influent color of 900 Pt‐Co units and absorbance values of 24.8, 18.11, and 18.5 m^−1^ at 436, 525, and 620 nm, respectively, demonstrated substantial improvement in performance after ozonation at a flow rate of 0.042 m^3^/h and contact times of 5 and 10 min. These results highlight the potential of ozonation not only in achieving regulatory discharge limits but also in enhancing overall environmental safety (Morrison et al. 2023). The integration of coagulation–precipitation/ozonation treatment has been reported to yield better results compared with single‐stage treatment systems. However, this combined approach resulted in low COD removal efficiency (Li et al. 2025). To enhance both color and COD removal, a higher dosage of ozone and coagulant is often required, which may raise operational costs and potentially lead to the formation of toxic by‐products from biodegradable substances during the ozonation process.

Overall, ozonation offers rapid color removal, detoxification, and improved biodegradability, particularly as a pretreatment before biological treatment. Nevertheless, challenges such as incomplete mineralization, formation of low‐molecular‐weight by‐products, and the energy demand of ozone generation must be considered in full‐scale applications (Hu et al. 2019; Rekhate et al. 2025).

### 6.3 UV Advanced Oxidation Processes (UV‐AOPs)

AOPs such as UV/H_2_O_2_, UV/O_3_, and UV/Cl are increasingly utilized for the treatment of textile wastewater due to their ability to degrade a wide range of pollutants (Gora et al. 2024; Khajouei et al. 2022). The UV/H_2_O_2_ process involves the photolysis of hydrogen peroxide under UV light to generate hydroxyl radicals (˙OH), which are highly reactive and capable of oxidizing various contaminants (Kumari and Kumar 2023). This method is well‐established and broadly effective; however, it is often associated with higher operational costs due to the need for considerable amounts of H_2_O_2_, as well as the potential formation of oxidation byproducts (Hubner et al. 2024). In contrast, the UV/Cl process employs chlorine in combination with UV light to produce chlorine‐based radicals, offering an alternative oxidation pathway with distinct reaction kinetics and byproducts (Moore et al. 2023). Primarily, the lower cost of chlorine compared with hydrogen peroxide suggests that UV/Cl may be more cost‐efficient; nonetheless, the formation of potentially harmful chlorinated byproducts and residual chlorine necessitates further investigation (Mackey et al. 2023). Several water matrix parameters, including pH, temperature, and the presence of competing substances, all influence the effectiveness of AOPs (Asghar et al. 2022). Given the complex composition of textile effluents, UV/H_2_O_2_, UV/O_3_, and UV/Cl present viable treatment options. AOPs may be less effective compared with ozonation for some treatment goals because they were more strongly influenced by inhibitory contaminants and physicochemical factors (e.g., pH) in real, undiluted wastewater (Bilińska et al. 2016). However, the selection between these technologies should be guided by a comprehensive assessment of treatment goals, operational costs, and the environmental impact of the generated byproducts.

Complementary oxidation technologies, such as solar photo‐Fenton systems, have shown promise in addressing the limitations (Ribeiro et al. 2017). For instance, Starling et al. (2021) demonstrated the effectiveness of persulfate‐mediated solar photo‐Fenton treatment (solar/Fe/S_2_O_8_^2−^) for the improvement of wastewater effluents at near‐neutral pH. Operating under solar irradiation (30 W m^−2^) with intermittent iron dosing, the process achieved over 60% removal of dissolved organic carbon (DOC) and substantial reductions in color, toxicity, and recalcitrant organic pollutants (V. M. Starling et al. 2021). These findings underscore the potential of persulfate‐based photo‐Fenton processes as viable and cost‐effective alternatives or complements to ozonation for achieving efficient pollutant degradation and detoxification in textile wastewater treatment.

### 6.4 Photocatalysis

Photocatalysis has been widely explored at the lab scale because of its ability to effectively eliminate a wide range of dyes and organic contaminants from industrial wastewater (Adeola et al. 2022; Khader et al. 2024). Although UV light supply and catalyst recovery are energy‐intensive, photocatalysis has the potential to be an eco‐friendly approach that does not generate secondary pollutants if complete degradation is achieved (Khader et al. 2024). Photocatalysis has demonstrated significant potential at the laboratory scale in addressing various wastewater treatment challenges, targeting dyes, hydrocarbons, insecticides, and microorganisms, and reducing harmful metal ions in textile wastewater, achieving up to 80%–90% treatment efficiency for many parameters (Fard et al. 2024). While specific UV dosages are not always reported, exposure times and intensities suggest that effective degradation typically occurs within a UV dosage range of 100–2000 mJ/cm^2^ (Choi et al. 2025; Cooper et al. 2010). This range varies based on factors such as catalyst type, dye concentration, and reactor designs. The efficiency of photocatalysts is influenced by factors such as catalyst morphology, electron–hole pair dynamics, pH, temperature, and light intensity, making parameter optimization essential for improved performance of chemical treatment processes (Lee et al. 2023).

The photocatalytic processes reported in the literature utilize various light sources such as a 500 W mercury lamp (λ < 420 nm) (Jiang et al. 2022), a 300 W xenon lamp (Huang et al. 2022), and solar light (Wang et al. 2017), along with several metallic and carbonaceous composites as catalysts. The first generation of metal oxide‐based photocatalysts, such as ZnO and TiO_2_, has been widely explored in the laboratory for textile wastewater treatment; however, limitations such as over‐dependence on UV light and incomplete degradation of textile dyes have been reported (Bopape et al. 2024). This has led to the development of second and third‐generation metal oxides for photocatalytic applications. These newer generations, such as ZrO_2_, FeVO_4_, GO/WS_2_/Mg‐doped ZnO, g‐C_3_N_4_/FeVO_4_, offer enhanced charge separation, faster kinetics, visible light utilization, immobilization technology, and superior degradation efficiency (Kumari et al. 2025). Catalyst shape and specific surface area also play critical roles in improving the efficiency of this chemical treatment method (Akinyemi et al. 2024; Bernard et al. 2021).

The development of materials to serve as photocatalysts also requires improvement in areas such as stability and durability, which are critical for their long‐term performance and cost efficiency, particularly in wastewater treatment applications (Fouda‐Mbanga et al. 2024; Thakur et al. 2024). For example, photocatalyst corrosion resistance, which refers to the material's ability to withstand degradation in corrosive environments, is still a major concern (Li et al. 2022). Corrosion in photocatalysts can be caused by chemical and electrochemical reactions, leading to material dissolution or catalytic deactivation (Dimitropoulos et al. 2024). Recent advancements have focused on improving the stability of catalysts to mitigate photo‐corrosion while maintaining their photocatalytic efficiency (Ning and Lu 2020; Toe et al. 2019). In 2023, Warren et al. investigated the doping of ZnO with 1% and 2% Co, Ni, or Cu salts, significantly enhancing its stability by reducing zinc ion leaching under light exposure. However, this doping process also led to reduced photocatalytic activity due to alterations in energy levels and band gap reduction (Warren et al. 2023).

While photocatalysis is a promising technology for textile wastewater treatment, further advancements are needed to enhance its efficiency. Future research should focus on maximizing the use of natural sunlight or low‐energy artificial light sources (e.g., light‐emitting diodes) to improve economic feasibility, investigating molecular‐level mechanisms to optimize degradation pathways, and improving reactor design through enhanced catalyst efficiency and mathematical modeling.

### 6.5 Electrochemical Advanced Oxidation Processes (EAOPs)

EAOPs utilize electric current to generate reactive species that degrade dyes and other organic pollutants (Alkhadra et al. 2022). Processes such as electrooxidation and electrocoagulation (EC) offer efficient treatment alternatives with lower chemical consumption than many conventional treatment technologies. However, these processes may still require supporting electrolytes, pH‐adjusting agents, or electrode‐conditioning chemicals to optimize treatment performance and conductivity (Etafo et al. 2025). Electrochemical techniques offer several advantages over other AOPs, including relatively mild operating conditions and lower operational costs (Rodríguez‐Peña et al. 2024). Additional merits include the ability to enhance treatment effectiveness by employing both cathodic and anodic processes, which can be automated (Esplandiu et al., 2024). In electrochemical treatments, current densities typically range from 10 to 25 mA cm^−2^, with treatment durations between 10 and 60 min and flow rates around 10 L h^−1^ in continuous systems. The impact of process conditions highlights the importance of optimizing flow rate, energy input, and treatment time to maximize pollutant removal efficiency in textile effluent treatment.

Boron‐doped diamond (BDD) anodes, known for their high stability, wide potential window, and degradation efficiency, have emerged as a particularly promising technology for treating real textile wastewater (Frontistis et al. 2017; Karim et al. 2021). In such applications, BDD anodes are often employed in undivided cells, where both the anode and cathode are housed in the same reaction chamber without a separating membrane (Divyapriya and Nidheesh 2021). This design avoids the costs and inefficiencies associated with membrane‐based (divided) systems, which typically require ion exchange membranes to separate oxidation and reduction reactions. By eliminating the membrane, undivided cells simplify system design, reduce maintenance requirements, and lower operational costs. Moreover, the unique properties of BDD anodes minimize undesirable side reactions, making them well‐suited for undivided setups (Chen et al. 2003; Martínez‐Huitle and Brillas 2021). This enhances the practicality, scalability, and overall economic feasibility of electrochemical treatment for industrial wastewater applications. However, most prior investigations have targeted high‐conductivity wastewater, leaving low‐conductivity textile effluents underexplored. The efficiency of BDD‐based electrochemical systems can be significantly reduced in low‐conductivity wastewater due to limited current flow and mass transfer limitations (Miao et al. 2022; Pérez et al. 2017). Advanced electrodes, such as BDD, titanium suboxide, or doped metal oxides, play a pivotal role by enabling high overpotential for oxygen evolution and minimal side reactions (Xiao et al. 2023). However, innovation remains needed in electrode design to enhance conductivity, stability in corrosive conditions, and resistance to fouling by dye intermediates and salts commonly present in textile effluents. Additionally, energy consumption can become substantial when treating large volumes or dilute effluents, impacting overall process sustainability. To address these research gaps, future work should evaluate the feasibility of electrochemical methods for low‐conductivity wastewater while minimizing the use of supporting electrolytes. Such advancements could make electrochemical approaches a viable, eco‐friendly, and cost‐effective solution for treating textile industry effluents.

### 6.6 Regeneration and Reuse of Materials in Textile Wastewater Treatment

Sorbent and photocatalyst regeneration is an essential process to restore the catalytic or adsorptive functionality of deactivated or contaminated photocatalysts and adsorbents (Khan et al. 2024). It also promotes the sustainability concept through reuse, regeneration, waste miniaturization, and operational cost reduction (Bockenstedt et al. 2021). Since photocatalysts and adsorbents interact with pollutants, effective regeneration strategies are crucial for maintaining their efficiency in several cycles of pollutant degradation or adsorption (Fouda‐Mbanga et al. 2024). Several studies have explored regeneration strategies to enhance photocatalyst stability and reusability. Miranda et al. explored the use of TiO_2_ photocatalysts immobilized on the glassy substrate for the degradation of fluorine‐containing contaminants over four cycles (Miranda‐García et al. 2014). Regeneration using H_2_O_2_/UV or calcination was effective in restoring the photocatalytic activity of the material. Similarly, Kim et al. explored the potential of TiO_2_‐coated zeolite as a photoregenerative adsorbent for volatile organic compound filters. The study demonstrated a photoregeneration efficiency exceeding 90% during the initial two cycles of regeneration under UV illumination (Kim et al. 2022). Compared with pristine zeolite filters, TiO_2_/zeolite exhibited superior regeneration efficiency, suggesting its potential for UV‐regenerative air filtration systems. The filter maintained > 60% efficiency over five cycles of regeneration and reuse, reinforcing its applicability as a reusable photoregenerative adsorbent. Aside from the use of H_2_O_2_/UV or calcination, other advanced regeneration approaches, such as the formic‐acid‐mediated regeneration method, have also been developed with good regenerative capabilities (Yin et al. 2022).

### 6.7 Techno‐Economic Analysis of Chemical Treatment Processes

#### 6.7.1 Coagulation and Flocculation

From a techno‐economic perspective, coagulation–flocculation may offer lower capital investment and simpler operation than other options for textile wastewater treatment (Hoa and Hue 2018). Some natural coagulants, such as alginate and chitosan, have unit prices of US$12 and US$19 kg^−1^ (Saranya et al. 2014). By contrast, alum prices range between US$0.30 and 0.50 kg^−1^, with reported volumetric water treatment costs varying widely between US$0.05 and 1.5 m^−3^ (Keeley et al. 2014; Kurniawan et al. 2020). Literature indicates that natural coagulants can be either more or less expensive than chemical alternatives, depending on the type, local availability, and optimum dosage. For example, calcium lactate (US$0.009 m^−3^) and tannic acid (US$0.004 m^−3^) had higher water treatment costs than polyaluminium chloride (US$0.0017 m^−3^) and ferric chloride (US$0.0024 m^−3^) (Nazari‐Sharabian et al. 2020). Similarly, chitosan treatment cost was reported as US$0.025 m^−3^ (Kangama et al. 2018), while Moringa‐based treatment is US$0.042 m^−3^ (Megersa et al. 2018). In contrast, other natural coagulants like *Azadirachta indica A. Juss* were more expensive (US$ 1.73 m^−3^) compared with alum (US$0.56 m^−3^) in urban wastewater applications (Thirugnanasambandham and Karri 2021). Emerging bio‐coagulants and natural polymers may further improve sustainability and cost‐effectiveness by minimizing sludge toxicity and reliance on metal salts (Diver et al. 2023).

EC systems can reduce reagent costs, though this is offset by higher electricity consumption and the cost of electrodes. Unlike conventional chemical coagulation systems (e.g., alum or ferric based), which can be 3.2 times more expensive than EC (Bayramoglu et al. 2007; Espinoza‐Quiñones et al. 2009). The operating cost of the EC process depends primarily on energy consumption, electrode dissolution, and electrolyte or pH‐adjusting chemical use (Syam Babu et al. 2019). The overall operating cost of EC can be expressed as the sum of the cost contributions from these parameters, where specific energy consumption typically ranges between 1 and 2 kWh m^−3^ (Lakshmi et al. 2013; Miklos et al. 2018). Treatment costs vary with wastewater type, electrode configuration, and scale. For example, Pirkarami and Olya (2017) estimated US$1.86 m^−3^ for dye removal using EC (Pirkarami and Olya 2017), while Bazrafshan et al. (2015) reported US$1.7 m^−3^ for tannery effluent, compared with US$3.5 m^−3^ using conventional coagulation (Bazrafshan et al. 2015). Pilot‐scale EC systems treating paper‐mill wastewater achieved costs between ≈US 0.07–0.11 m^−3^, reflecting energy consumptions of 0.48–0.62 kWh m^−3^ (Perng et al. 2007). Similarly, a pilot EC unit designed for Canadian remote communities achieved significant color (54%) and DOC (37%) reductions at costs below US$0.27 m^−3^ (Perng and Wang 2016).

#### 6.7.2 Ozonation

The main economic concerns of ozonation can be attributed to the energy demand for ozone generation, oxygen or air feed, and maintenance of the generator units. Capital cost is primarily associated with the ozone generator, gas–liquid contactors, and off‐gas destruction systems, while operating costs were dominated by electricity consumption (Amaya‐Santos et al. 2025). Despite these requirements, ozonation was found to offer a favorable balance between treatment efficiency and overall cost, particularly when high color removal and toxicity reduction are prioritized (Castañeda‐Retavizca et al. 2025). The operating cost of ozonation was estimated at approximately US$1.3 m^−3^ of treated wastewater, based on pilot‐scale energy and reagent consumption. The electric energy consumption for ozonation typically ranges from 8 to 15 kWh m^−3^ of treated wastewater, depending on the influent COD and dye concentration. At an average industrial electricity rate of US$0.10 kW^−1^ h^−1^, this corresponds to an energy cost of approximately US$0.80–1.50 m^−3^ for ozone generation, while oxygen or air supply and equipment maintenance contribute an additional US$0.10–0.20 m^−3^. Consequently, the total operating cost of ozonation generally falls between US$1.0 m^−3^ and US$1.3 m^−3^ of treated textile wastewater (Bilińska et al. 2016). This cost level positions ozonation as moderately expensive compared with low‐energy biological systems, whose typical operational costs for activated sludge or membrane bioreactor (MBR) setups range from US$0.09 to 0.44 m^−3^ (Verma et al. 2012), but more cost‐competitive than reagent‐intensive AOPs, such as UV/H_2_O_2_ and photo‐Fenton processes. Photo‐Fenton processes, depending on H_2_O_2_/Fe dosages and irradiation source, range widely from US$1.3 m^−3^ under solar conditions to more than US$20–22 m^−3^ when artificial light is used (Santos‐Juanes Jorda et al. 2011; Starling, Dos Santos, et al. 2017). In contrast to these other AOPs, ozonation avoids the use of chemical reagents that generate sludge, thereby reducing downstream disposal costs and operational complexity. Furthermore, ozonation maintained high performance even when applied directly to industrial effluents containing auxiliary compounds, surfactants, and salts, factors that often inhibit other AOPs, thus offering an additional economic advantage by reducing the need for pre‐treatment (Mousset et al. 2021). The process also proved effective in toxicity abatement, a feature that may translate into indirect cost savings by lowering regulatory risks and minimizing the need for post‐treatment/tertiary disinfection.

From a process engineering perspective, ozonation also benefits from modular scalability and relatively straightforward operation, making it suitable for retrofitting existing treatment plants. However, the total cost remains dependent on ozone dose, initial COD, effluent composition, and local electricity prices, which can influence the economic favorability relative to competing technologies (Aber et al. 2025; Leontieff et al. 2025).

#### 6.7.3 Advanced Oxidation Processes (AOPs)

AOPs are powerful treatment technologies used in textile wastewater remediation to degrade recalcitrant dyes and organic pollutants through the in situ generation of highly reactive species, primarily hydroxyl radicals (Khan et al. 2023). Common AOPs include UV/H_2_O_2_, Fenton and photo‐Fenton processes, electro‐Fenton, photocatalysis, and persulfate‐based oxidation (Hubner et al. 2024). Of these, only UV/H_2_O_2_ is used at the full scale for any form of municipal or industrial wastewater treatment. UV combined with hypochlorite (UV/chlorine) is also used for municipal and industrial water treatment (Mackey et al. 2023). Although some costing information for UV/H_2_O_2_ and UV/chlorine processes is available in the municipal water and wastewater literature (e.g., Mackey et al. 2023), no information about the cost of these processes for textile wastewater treatment was found in the literature reviewed for this study.

A preliminary cost assessment conducted by Starling et al. (2017) examined the economic feasibility of AOPs for textile wastewater treatment and reuse. The study compared conventional and ferrioxalate photo‐Fenton systems operated under UV and visible light sources for real dye‐house effluents and found that treatment costs were estimated at approximately US$0.91–1.07 m^−3^ respectively for the two systems (Starling, Castro, et al. 2017). Although described as preliminary, this analysis provided valuable insights into the relative operational costs of AOPs for industrial wastewater treatment, emphasizing the potential for cost‐effective water reclamation in textile applications. However, the study did not include a comprehensive techno‐economic evaluation encompassing capital, maintenance, and life‐cycle costs, thereby limiting its applicability for large‐scale economic projections. In a related study, Starling's group evaluated a semi‐pilot scale compound parabolic collector (CPC) reactor implementing the solar photo‐Fenton process for treating recalcitrant textile wastewater (Starling, Dos Santos, et al. 2017). Although the system achieved ~96% removal of DOC and 99% of absorbance under optimal conditions, no explicit quantitative cost analysis or techno‐economic breakdown was provided within this work.

In the absence of costing information, the electrical energy per order (E_EO_) metric can be used to evaluate and compare the energy efficiency of UV‐AOPs (Miklos et al. 2018). E_EO_ (kWh/m^3^/order) can be calculated using Equation (2) below:

(2) $$E_{\mathrm{EO}}=\frac{P\;x\;t}{\mathrm{Vx}\;\log_{10}\left(\frac{C_{o}}{C_{f}}\right)}$$

where P is lamp power (kW), t is irradiation time (hours), V is volume of water treated (m^3^), $C_{o}$ is initial contaminant concentration, and $C_{f}$ is final contaminant concentration (Keen et al. 2018). Several factors influence the E_EO_ value. The wastewater composition may also affect E_EO_, as constituents like natural organic matter and bicarbonate can act as ˙OH scavengers, altering the steady‐state ˙OH concentration (Dong et al. 2010). Additionally, the nature of the contaminant, such as its reactivity with ˙OH (i.e., second‐order rate constant) and its ability to undergo direct photolysis, significantly influences the degradation rate and thus the E_EO_ (Yuan et al. 2009). Reactor design and hydraulics, which include UV lamp type, reactor geometry, and mixing efficiency, further impact the irradiance distribution and contact time, affecting energy consumption (Keshavarzfathy and Taghipour 2019; Sultan et al. 2019). Very few of the studies that were reviewed calculated E_EO_, and most papers did not report all the necessary parameters required to estimate energy consumption and associated cost. To promote consistency, comparability and field/full‐scale applications, it is recommended that E_EO_ and/or the key parameters used to calculate it be reported in a standard format. It is recommended that other parameters should be included in scientific reports/research articles, such as H_2_O_2_ concentration used, the probe compound for ˙OH assessment along with its concentration and reaction rate constant, detailed water matrix characteristics like total organic carbon (TOC) and alkalinity, and observed reaction rates, both overall and due to direct photolysis (Hwang et al. 2020; Keen et al. 2018).

#### 6.7.4 Photocatalysis

Photocatalysis is distinct from UV and solar light‐driven AOPs because it incorporates a reusable catalyst, which introduces new cost elements while reducing or eliminating oxidant costs. Additionally, unlike UV AOPs, photocatalysis has not been widely adopted at the full scale for municipal or industrial water treatment.

A cost analysis by Asha et al. compared GAC–TiO_2_ photocatalytic treatment with UV–GAC, UV‐only, and GAC‐only methods, demonstrating that the hybrid UV–GAC–TiO_2_ system achieved complete contaminant removal within minutes and the lowest operational cost per unit mass removed, owing to synergistic adsorption–photocatalytic effects, enhanced radical generation, and significantly reduced treatment time (Asha et al. 2015). Energy consumption for treating wastewater over 240 min in UV and UV‐TiO_2_ systems was 0.524 kW h^−1^, costing US$47.55 and US$32.07 kg^−1^ of targeted contaminant removed, respectively. Using GAC‐UV systems significantly improved contaminant removal efficiency, increasing from 58% with UV alone to 100% with UV‐GAC, in just 120 min. The UV‐GAC system was more cost‐effective, reducing operational costs by 57%–71% compared with UV alone and UV‐TiO_2_ systems. However, GAC alone showed minimal effect. The UV‐GAC‐TiO_UU_ system achieved 100% contaminant removal in just 6 min, with the lowest operational cost of US$0.68 kg^−1^ of pollutant removed. This system is more economical than UV‐only and GAC‐only due to GAC‐TiO_2_’s higher specific surface area, porous structure, and active sites for radical production. Additionally, varying flow rates (60, 80, and 100 mL min^−1^) impacted energy consumption and contaminant removal efficiency, with the highest removal rate observed at 60 mL min^−1^. Overall, UV‐GAC‐TiO_2_ is a cost‐effective and efficient method for treating wastewater in continuous photocatalytic operations.

In a recent study by Baaloudj et al. (2022) on photocatalytic systems for pollutant degradation, the total fabrication and implementation cost of the proposed system was estimated at US$1520 (US$4000 m for a total volume of 0.38 m^3^). This cost included the construction of 6 mm acrylic photocatalysis and sedimentation units. The mechanical and electrical equipment were valued at US$950 (US$2500 m^−1^ for a total volume of 0.38 m^3^). The preparation cost for the catalyst, Bi_12_TiO_20_, was calculated at US$1.74 m^−3^ based on local supplier prices in Algeria. Energy consumption costs for 12 Philips energy‐saving lamps were calculated based on a local electricity tariff of US$0.033 kw^−1^ h^−1^. The cost of technicians, chemists, and workers' salaries was estimated at US$43.4 m^−3^. Additional costs, such as sludge handling, were considered to be US$2 m^−3^. Reusing the regenerated catalyst resulted in a 30% reduction in the system's operating costs, with further energy savings from relying on sunlight in the morning (Baaloudj et al. 2022).

## 7 Biological Treatment Processes for Textile Wastewater

Biological treatment is an established wastewater treatment technology that provides a potentially eco‐friendly and cost‐effective approach for contaminant removal through the metabolic activities of microorganisms such as bacteria, fungi, algae, and yeasts (Ghasemian‐Dastjerdi et al. 2026; Wani et al. 2026). In the context of textile wastewater treatment, the novelty lies not in the technology itself, but rather in its application for the remediation of increasingly complex textile effluents containing recalcitrant dyes, auxiliary chemicals, and emerging contaminants that are often resistant to conventional treatment processes. Advanced biological technologies like microbial fuel cells (MFC), though well established in the peer‐reviewed literature, remain at the initial stages of real‐world implementation.

### 7.1 Biological Treatment Processes

Biological treatment of textile wastewater has been investigated using a diverse range of microbial and bio‐based processes, including anaerobic and aerobic reactors, activated sludge systems, MBRs, anaerobic digestion, bacterial consortia, fungal treatments, and algal–bacterial systems (Table 3). These approaches rely on the metabolic activity of microorganisms to remove dyes, organic matter, nutrients, and other contaminants, offering a potentially sustainable and cost‐effective alternative to physicochemical treatment methods.

**TABLE 3:** Biological treatment processes and performance metrics reported in past textile wastewater treatment studies.

Biological processes	Target compounds	Optimal conditions	Efficiency (%)	Research scale	References
Anaerobic–aerobic sequential reactor	Color, COD, nutrients	Anaerobic: pH 7.8, Hydraulic retention time (HRT) 9 days; aerobic: HRT 3 days	Color: 68%; COD: 71%; Nitrate & Ammonia: 100%	Lab scale	(Bidu et al. 2023)
Up‐flow anaerobic sludge blanket	Color, COD	HRT 24 h; no carbon supplementation	Color: 97%; COD: 90%	Lab scale	(Somasiri et al. 2008)
Bacterial consortium	COD, Dyes	37°C; 32 h reaction time	COD: 98%; Dye: 88%	Lab scale	(Deng et al. 2020; Rathour et al. 2019)
*Aspergillus niger* (fungal biosorbent)	Acid dyes	pH adjustment; batch absorption	Dye removal: 98%	Lab scale	(Li et al. 2019)
Fungal treatment (white‐rot fungi)	Reactive and disperse dyes	pH 4.5–5.5, temperature 28°C–32°C, incubation 5–10 days	70%–100% (color), 40%–70% (COD)	Lab scale	(Kaushik and Malik 2009; Pointing 2001)
Algal‐bacterial systems	Nutrients, dyes, heavy metals	Light: 12 h photoperiod, pH 7.5–8.5, temperature 25°C–30°C	60%–85% (color), 70%–90% (nutrients)	Lab scale	(Rawat et al. 2011)
Membrane bioreactor (MBR)	Dyes, COD, TSS	MLSS: 8000–12,000 mg/L, pH 6.5–8, temperature 25°C–30°C	> 90% (COD, TSS), 85% (color)	Pilot scale	(Hai et al. 2006)
Anaerobic digestion	Azo dyes, BOD	pH 6.5–7.5, temperature 35°C (mesophilic), HRT 20–30 days	60%–80% (Color), 50%–70% (COD)	Lab scale	(dos Santos et al. 2007)
Activated sludge process	Dyes, COD, BOD, surfactants	pH 6.5–8, temperature 25°C–35°C, HRT 6–24 h	70%–90% (COD), 80% (Dyes)	Lab scale	(Hai et al. 2007)
Membrane bioreactor	Reactive Red 390 dye, COD	pH: ~7.5, Temperature: 20°C ± 1°C, HRT: 16.9, SRT: 30 days, MLSS: 6000–10,000 mg/L, DO: > 3 mg/L, Flux: 5.01 L/m^2^·h	COD removal: 93.1%–98.5%; Color removal: 87.1%–89.5%	Lab scale	(Sari Erkan et al. 2020)

Abbreviations: BOD: biochemical oxygen demand; COD: chemical oxygen demand. HRT: hydraulic retention time; MLSS: mixed liquor suspended solids. TSS: total suspended solids.

The effectiveness of microbial‐driven water treatment technology is largely impacted by the adaptability and enzymatic activities of microorganisms employed (Kaur et al. 2024; Zheng et al. 2024). Several studies have revealed that various bacteria can achieve high decontamination rates of dyes, i.e., *Proteus mirabilis* LAG removed 84% of Reactive Blue 13 (Olukanni et al. 2010), while *Kocuria rosea* achieved 100% removal of 50 mg/L Methyl Orange (Parshetti et al. 2010). In the 1970s, bacteria such as *Bacillus subtilis* and *Aeromonas hydrophila* displayed unique abilities for azo dye degradation, which has now been optimized to over 99% efficiency by recent advancements using anaerobic MBRs (Singh et al. 2024; Yang et al. 2018).

Considerable research efforts have focused on optimizing biological systems and developing novel microbial consortia capable of enhancing contaminant degradation and treatment efficiency (Rendón‐Castrillón et al. 2024). Bacterial consortia have demonstrated high efficiencies with 99.26% Red 198 azo dye degradation in 72 h, previously reported (Eslami et al. 2019).

Supplementation with nutrients and other factors to improve biodegradation has also been explored in past literature. Bacteria such as *Aeromonas veronii* GRI have shown promising results in degrading Methyl Orange when supplemented with sucrose, yeast extracts, and biosurfactants like *Bacillus subtilis* SPB1 lipopeptides (Mnif et al. 2016). These biosurfactants improve the degradation process by enhancing cell permeability and accelerating enzyme‐mediated reactions (Markam et al. 2024). Enzymes such as azo reductase, laccase, and peroxidases facilitate the breakdown of complex textile compounds, thus optimizing the process (Das et al. 2023). Moreover, optimal conditions such as pH (6.0–10.0), temperature, and the presence of electron donors like glucose also play a vital role in ensuring optimal treatment efficiency (Logan et al. 2023; Poothong et al. 2024).

Sequential anaerobic–aerobic systems have been successfully implemented on a pilot scale, showing significant reductions (> 90%) in color and COD in real textile wastewater systems (Kozak et al. 2021). In general, biological treatment processes often require long retention times and are highly sensitive to fluctuations in pH, temperature, and toxic compound concentrations. Additionally, the incomplete mineralization of complex dyes and the potential accumulation of intermediate metabolites may limit treatment effectiveness and environmental safety.

### 7.2 MFCs

**FIGURE 6:**
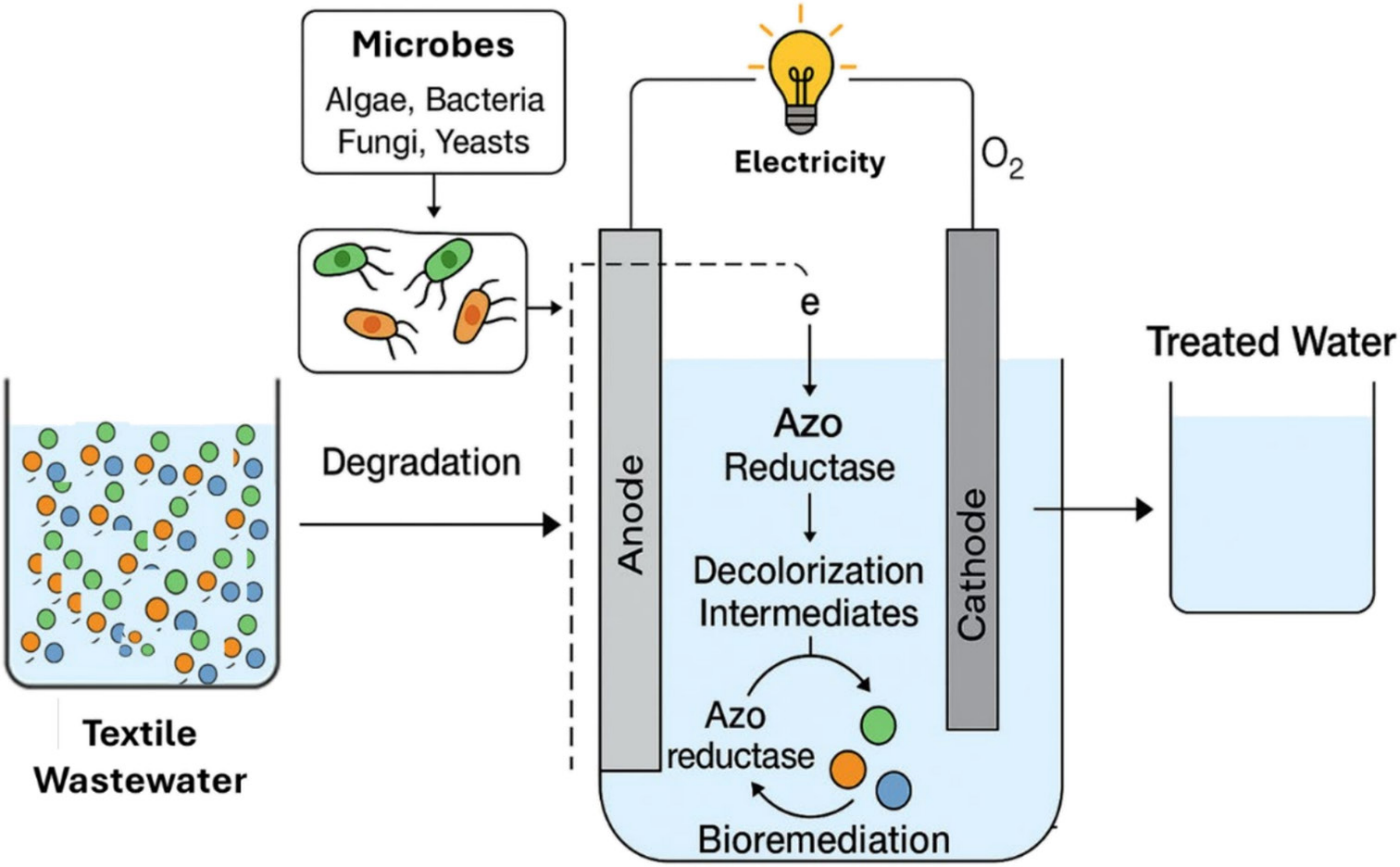
Schematic representation of microbial fuel cell (MFC)‐assisted bioremediation of textile wastewater. Electroactive microorganisms (e.g., bacteria, fungi, algae, yeasts) degrade complex dye compounds via enzymatic activity (e.g., azo reductase), producing decolorization intermediates and generating electrons that flow to the cathode to produce bioelectricity.

MFCs offer a dual advantage of wastewater treatment and bioelectricity generation (Malik et al. 2023). These systems leverage electrochemically active bacteria to degrade dyes while generating electrons for power generation (Bazina et al. 2023). Advancements in air‐exposed single‐chamber MFCs and dual‐MFC setups have demonstrated significant COD removal (up to 98%) and efficient textile dye degradation (> 98.7%). These demonstrate the potential of integrating biological treatment with energy recovery (Figure 6), providing a sustainable solution for textile wastewater treatment (Fatima et al. 2021). In a recent study, a single‐chamber anti‐gravity flow MFC was developed and used to investigate the treatment of azo dyes, Acid Orange 7, and Reactive Green 19, in both single and binary systems (Tan et al. 2022). The study demonstrated high COD removal (~87%) and effective dye decolorization, although bioelectricity output declined with increasing initial dye concentrations. The complexity of binary dye systems, due to electron competition and intermolecular interactions, posed greater treatment challenges than single‐dye setups. Sulfate and its reduction byproducts, particularly sulfide, influence degradation performance, while quinoid‐based redox mediators may promote electron transfer and pollutant breakdown (Jin et al. 2022; Tan et al. 2021; Zhang, Cheng, et al. 2023).

Notably, it has been reported that some decolorization intermediates contributed to autocatalytic enhancement of both dye removal and electricity production in MFCs (Tan et al. 2022; van der Zee et al. 2000), although their mechanisms remain underexplored. This work underscores the potential of MFCs for real textile wastewater treatment while highlighting the need for further research on multicomponent pollutant systems and redox‐active intermediates. Despite the potential of MFC systems, they face limitations such as low power output and scalability challenges for industrial applications (Tan et al. 2022; Teoh et al. 2023). Moreover, electrode fouling, internal resistance, and the need for strict environmental control can hinder long‐term stability and performance.

### 7.3 Comparison of the Effectiveness of Biological Treatment Processes for Textile Wastewater Treatment

Table 3 highlights the significant potential of biological treatment technologies for the remediation of textile wastewater, particularly for the removal of dyes, color, COD, BOD, nutrients, and suspended solids. Reported treatment efficiencies generally range from 60% to nearly 100%, depending on the biological system, wastewater characteristics, and operating conditions. Conventional biological processes, including activated sludge, anaerobic digestion, and anaerobic–aerobic sequential reactors, demonstrate effective removal of organic matter and nutrients, while specialized microbial, fungal, and algal systems show enhanced capabilities for dye degradation and decolorization. MBRs consistently achieve some of the highest COD, TSS, and color removal efficiencies due to the integration of biological degradation and membrane separation. Despite these promising results, most studies have been conducted at laboratory scale, with just one pilot‐scale investigation. Consequently, further research is required to evaluate long‐term operational stability, treatment of complex real textile effluents, sludge management, and economic feasibility to facilitate broader industrial‐scale application.

### 7.4 Technoeconomic Analysis of Biological Treatment for Textile Wastewater Treatment

Although techno‐economic assessments of biological treatment technologies for textile wastewater remain relatively scarce, several studies have reported operational costs for full‐scale implementation. For example, Yang et al. (2020) compared conventional activated sludge (CAS), MBR, and moving bed biofilm reactor (MBBR) systems for textile wastewater treatment. All reported costs were inflation‐adjusted to January 2026 US dollars using the CPI and applicable currency exchange rates. The reported operating costs were approximately US$1.55 m^−3^ for CAS, US$1.03 m^−3^ for MBR, and US$1.16 m^−3^ for MBBR (Yang et al. 2020). Electricity consumption represented a major cost component, accounting for approximately US$0.20, US$0.56, and US$0.10 m^−3^ for CAS, MBR, and MBBR, respectively. While MBR achieved the highest treatment efficiency, MBBR was identified as the most economically attractive option because it delivered comparable treatment performance with substantially lower capital investment. The estimated capital expenditure for full‐scale implementation was approximately US$402,600 for MBR membrane installation compared with US$127,050 for MBBR carrier media, representing a capital cost reduction of approximately 68% for the MBBR configuration. These findings suggest that biological treatment technologies can achieve treatment costs in the range of US$1–2 m^−3^ of textile wastewater, depending on reactor configuration, energy requirements, sludge production, and water reuse objectives (Yang et al. 2020).

Similarly, pilot‐scale investigations of MBR systems for textile wastewater treatment have highlighted membrane replacement, aeration energy demand, and fouling control as the primary contributors to operating costs (Deowan et al. 2019). Hybrid biological–membrane systems have also been evaluated from an economic perspective. For example, an MBR–NF process designed for textile wastewater reuse demonstrated favorable economic performance through water recovery and reduced plant footprint, with an estimated payback period of approximately 3 years under full‐scale operation (Li et al. 2020).

## 8 Emerging Technologies and Prospects in Textile Wastewater Treatment

Many recent studies have focused on emerging technologies for textile wastewater treatment, such as metal organic frameworks (MOFs), single‐atom catalysts (SACs), EAOPs, and nanobubble (NB) technology as alternatives to the technologies described in Sections 5 to 7.

### 8.1 MOFs and SACs

The catalytic potential of MOFs and SACs has garnered increasing attention for wastewater treatment due to their high surface area, tunable active sites, and enhanced light‐harvesting capabilities (Chaouiki et al. 2024; Wei et al. 2024). MOFs, with their porous crystalline structures and modular organic–metal architecture, offer a tunable catalytic system that facilitates efficient adsorption and degradation of organic micropollutants (Marinho et al. 2025). Their photocatalytic activity typically involves light‐induced generation of electron–hole pairs, followed by the formation of reactive oxygen species (ROS) such as hydroxyl (˙OH) and superoxide (˙O_2_^−^) radicals, which attack and mineralize contaminant molecules as previously described (Figure 5). However, MOFs often suffer from structural instability in aqueous media and limited charge separation efficiency, which can reduce their long‐term photocatalytic efficiency (An et al. 2024). On the other hand, SACs consist of isolated metal atoms dispersed on supports and exhibit exceptional catalytic activity and selectivity due to their unique electronic structures and maximal atom utilization (Wang et al. 2025). In textile effluent treatment, SACs enable efficient activation of oxidants and enhanced ROS production, facilitating rapid degradation of persistent pollutants under mild conditions (Li et al. 2024). Despite these advantages, challenges such as the scalability of synthesis, stability under irradiation, and potential toxicity of degradation byproducts must be addressed. Overall, while MOFs and SACs represent promising directions in photocatalytic remediation, further research into their durability, reusability, and integration with hybrid treatment systems is essential for their practical deployment in industrial‐scale textile wastewater treatment.

### 8.2 NB Technology

NB technology, which introduces gas‐filled cavities < 200 nm into aqueous solutions, offers promising mass transfer enhancements and oxidative potential due to bubble collapse‐induced generation of ROS (especially ˙OH and H_2_O_2_) (Jia, Farid, et al. 2023). In textile wastewater treatment, NB systems can facilitate degradation of organic contaminants, enhance flotation in pre‐treatment steps, and synergize with other oxidation processes such as ozonation or UV irradiation (Hutagalung et al. 2023; Satyam and Patra 2025). Mechanistically, the zeta potential and surface charge of NBs can also interact electrostatically with organic molecules, improving contact efficiency. Nonetheless, bottlenecks include the energy cost of NB generation, short bubble lifespan in complex environments, and lack of standardization in reactor design (Sakr et al. 2022). Furthermore, while laboratory‐scale results are promising, pilot‐scale implementations remain rare, and the influence of real effluent matrices (e.g., high TDS, surfactants, or complex organic mixtures) on NB behavior is not well‐understood and requires more research.

### 8.3 Hybrid Treatment Approaches

The integration of multiple treatment technologies has proven effective and may address the challenges of textile wastewater treatment (Asheghmoalla and Mehrvar 2024). It is also more in line with how textile wastewater is treated at the full scale. Physicochemical processes such as coagulation–flocculation effectively remove some contaminants but generate residues (e.g., backwash water), making them less cost‐effective unless integrated with other methods (Desta and Bote 2021). The integration of coagulation–flocculation with NF enhances effluent treatment, achieving > 98% efficiency by leveraging the strengths of both techniques (Sawadogo et al. 2024; Zheng et al. 2011). Similarly, AOPs that combine UV with multiple oxidants (e.g., O_3_/UV/H_2_O_2_) can improve pollutant degradation while minimizing microbial inhibition, ensuring that subsequent biodegradation is more effective (Demir‐Duz et al. 2022; Kumari and Kumar 2023). Pre‐treating textile effluents with coagulation–flocculation before oxidation treatment reduces turbidity and enhances treatment efficiency (Marques et al. 2025; Torres et al. 2019). The integration of multiple treatment approaches often optimizes treatment efficiency, reduces environmental impact, and enhances the feasibility of large‐scale textile wastewater remediation. While integrated treatment approaches offer improved efficiency and broader contaminant removal, their complexity can lead to higher capital and operational costs, particularly when multiple treatment units must be maintained and synchronized. Additionally, the compatibility of sequential processes is not always guaranteed, and the optimization of operational conditions across different units remains a technical challenge that can affect treatment consistency and scalability.

### 8.4 Research Gaps and Real‐World Implementation of Emerging Technologies

Despite the considerable progress achieved with emerging treatment technologies, several unresolved challenges must be addressed before large‐scale implementation can be realized. Long‐term material stability under complex textile wastewater conditions remains a major concern, particularly for MOFs and SACs that may undergo structural degradation, fouling, or loss of catalytic activity during prolonged operation (Oshani et al. 2025). The potential leaching of metals, ligands, nanoparticles, or degradation byproducts into treated water also raises environmental and human health concerns that require comprehensive risk assessment (Kholopo and Rathebe 2025). Furthermore, emerging treatment processes such as EAOPs and NB systems can be energy‐intensive, potentially limiting their economic feasibility and increasing their environmental footprint. Therefore, future studies should incorporate life‐cycle assessment (LCA), techno‐economic analysis, and environmental impact evaluations to determine the overall sustainability of these technologies.

## 9 Research Gaps and Future Research Opportunities for Textile Wastewater Treatment

Despite extensive past research on textile wastewater treatment at the laboratory scale, several research gaps and challenges still require attention, including the following:

1. Most past research has been limited to laboratory‐scale experiments focused on the removal of indicator dyes
2. There is only limited past research on the removal of important textile wastewater components such as salts, metals, and anthropogenic organic compounds (e.g., PFAS)
3. Most past studies have been conducted on single‐pollutant water matrices (e.g., indicator dye in purified water) or using simplified synthetic wastewater matrices that do not adequately represent real textile wastewater
4. Information reported in past studies is insufficient to conduct an effective comparison of the technoeconomic feasibility of different treatment processes.
5. LCA will be critical for identifying treatment systems that not only achieve high contaminant removal efficiencies but also minimize resource consumption, greenhouse gas emissions, secondary pollution, and operational costs over their entire service life.

**FIGURE 7:**
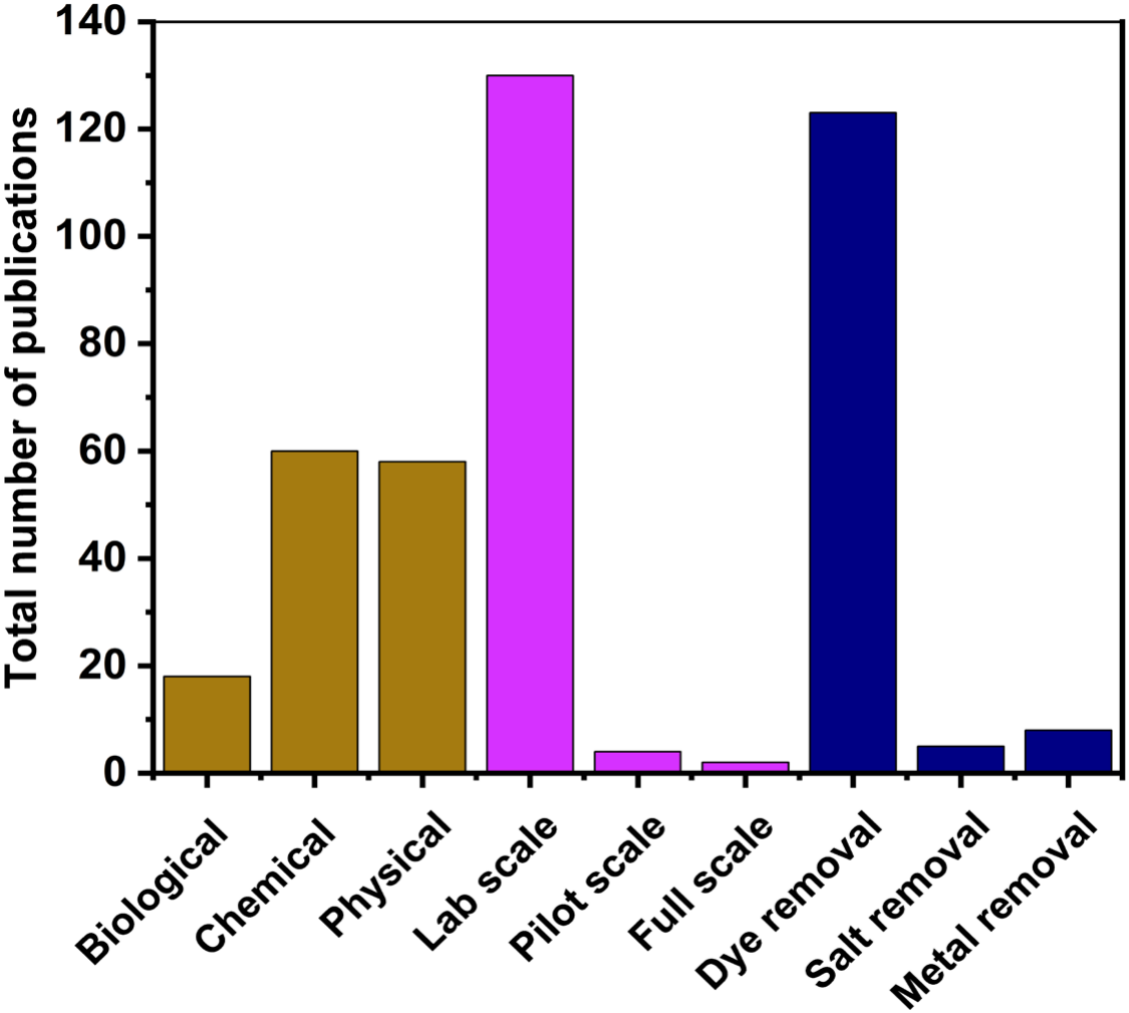
Number of studies published on the treatment of textile wastewater using physical, chemical, and biological methods between 2000 and 2025.

A key issue is scaling up emerging physicochemical and biological processes for real‐world applications. Although lab‐scale studies show high performance using NF, photocatalysis, AOPs, and MFCs, their transition to pilot or full‐scale use remains limited (Figure 7). Over 90% of the more than 120 studies that we reviewed were conducted at the lab scale, with less than 5% adopted at full scale. Most research also prioritizes dye removal over salt or metal decontamination (Figure 7). Major barriers to scale‐up include high energy demands, catalyst degradation, membrane fouling, and operational challenges in complex wastewater matrices (Satyam and Patra 2024; Zhang et al. 2024). Many laboratory‐scale textile wastewater treatment studies rely on synthetic wastewater containing selected dyes and salts at controlled concentrations. This approach improves experimental reproducibility and facilitates mechanistic investigations under well‐defined conditions. However, such simplified systems often fail to capture the variability and complexity of real textile effluents. Actual textile wastewater typically contains complex mixtures of dyes, finishing agents, surfactants, heavy metals, and suspended solids, with fluctuating pH, salinity, and organic loads that vary according to the production process and operating cycle (Saini et al. 2023). As a result, treatment processes such as adsorption, coagulation, and advanced oxidation often exhibit higher removal efficiencies (60%–99%) in synthetic wastewater due to the absence of competing species and lower turbidity. This may lead to an overestimation of their real‐world performance. In contrast, the presence of diverse contaminants and complex matrices in real effluents can hinder radical generation, block adsorption sites, and alter degradation kinetics. Furthermore, variable salt content and low conductivity of real effluents also may constitute a challenge compared with synthetic wastewater, where supporting electrolytes are often added. However, adding such chemicals in real‐world applications is economically and environmentally impractical, as it increases costs and risks of secondary pollutant formation (Hand and Cusick 2021; Najafinejad et al. 2023). Moreover, interactions between treatment methods and site‐specific hydrochemistry are not fully understood, leading to inefficiencies when scaling up to field applications.

Hybrid approaches, such as combining AOPs with biological methods or membrane systems with adsorption, require further research to enhance performance while minimizing costs and secondary waste. Pilot‐ and industrial‐scale testing of hybrid and emerging technologies in real settings is essential to assess long‐term feasibility, optimize operational parameters, improve efficiency, ensure accurate performance assessment, and reliable scale‐up of treatment technologies. For example, future research into adsorption and photocatalytic wastewater treatment processes should focus on developing efficient materials that retain their treatment capacity over multiple cycles, promoting sustainability and reducing material costs. Strategies include designing catalysts resistant to deactivation or incorporating protective measures to prevent fouling (Kuspanov et al. 2023). Extending the service life of catalysts and adsorbents and reducing replacement and waste‐handling costs remain priorities. Immobilizing catalysts on suitable substrates can enhance reusability with minimal efficiency loss (Xue et al. 2023), but further studies are needed to evaluate long‐term performance and maintain activity. Advanced research should also focus on improving photocatalytic reactor designs and pilot‐scale adsorption testing using continuous flow systems like Rapid Small Scale Column Tests (RSSCTs) to enhance performance. Optimizing reactor configurations to increase mass transfer and photon utilization can significantly boost efficiency (Liu, Li, et al. 2024). Maximizing sunlight use through systems that concentrate both direct and diffuse light can further improve photocatalytic effectiveness (Pérez and Bueno 2023).

In light of these research gaps, we recommend the following opportunities for future research:

- Evaluate existing and novel technologies for the removal of salts, metals, and other priority contaminants as described in the ZDHC standards (e.g., PFAS)
- Conduct experiments in water matrices more representative of textile wastewater.
- Explore hybrid treatment approaches that combine multiple promising technologies, such as AOPs followed by biological treatment or adsorption and membrane filtration
- Scale up promising technologies such as adsorption and photocatalysis, focusing on the practical aspects of the technology (e.g., fouling, service life, etc.)
- Report information required to evaluate the technical, economic, environmental, and social sustainability of technologies developed at the laboratory scale

Future studies should prioritize realistic bench‐scale experiments that bridge the gap between laboratory proof‐of‐concept and pilot‐scale implementation. This requires the use of real or well‐defined synthetic textile wastewater containing representative mixtures of dyes, salts, surfactants, finishing agents, and background organic matter at relevant concentrations. Such approaches are essential for generating performance data that are transferable to full‐scale systems and meaningful for techno‐economic evaluation.

For adsorption‐based processes using synthetic or natural adsorbents, future research should report parameters that enable estimation of volumetric treatment costs. In addition to adsorbent characterization, studies should include projected bulk material costs and scalability considerations, as well as operating conditions such as adsorbent dose, pH, contact time, and the influence of competing ions and auxiliaries common in textile effluents. Critically, equilibrium adsorption capacities should be complemented by dynamic performance data from fixed‐bed or column studies, including breakthrough curves and treated bed volumes under realistic wastewater conditions.

In coagulation and flocculation studies, greater emphasis should be placed on chemical consumption and sludge‐related metrics, which often dominate operating costs at scale. Future work should report coagulant type, purity, unit cost, optimal dosage ranges, and pH adjustment requirements. Performance evaluation should include both percentage and mass‐based removal of color, COD, turbidity, and suspended solids. Comprehensive sludge characterization, covering sludge yield (kg dry solids per m^3^ treated), water content, settling behavior, and dewatering requirements, is equally important. Energy consumption associated with mixing and pumping, as well as downstream sludge handling and disposal, should also be quantified to enable realistic cost assessment.

For AOPs, consistent reporting is particularly critical given the wide variability in energy and chemical demands across systems. Studies should clearly describe reactor configuration, oxidant type and dose, catalyst loading and recovery, and pH control requirements. Energy metrics should be reported in standardized units, including total energy consumption (kWh/m^3^) and, where applicable, EEO. Performance assessment should extend beyond decolorization to include COD and TOC mineralization, by‐product formation, and changes in effluent toxicity. The influence of salts, surfactants, and radical scavengers commonly present in textile effluents should be explicitly evaluated due to the matrix sensitivity of AOPs.

### 9.1 Recommendations for Standardized Techno‐Economic Reporting in Future Studies

To improve the comparability of adsorption and wastewater treatment studies, future investigations should adopt standardized approaches for reporting techno‐economic metrics. One useful parameter is the adsorption cost, defined as the cost associated with removing a unit mass of contaminant. This metric normalizes material and energy expenditures against adsorption capacity and facilitates comparison among adsorbents with different performances and production costs (Bajić et al. 2016). Future studies are encouraged to report adsorption costs using consistent units (e.g., US$ kg^−1^ or US$ g^−1^ contaminant removed) according to Equation (3).

(3) $$\text{Adsorption cost}\;\left(\frac{\mathrm{USD}\text{\$}}{g_{\text{contaminant}}}\right)=\frac{\left[\text{cost of}\;\mathrm{raw}\;\text{materials}\;\left(\frac{\mathrm{USD}\text{\$}}{g}\right)+\text{energy cost}\;\left(\frac{\mathrm{USD}\text{\$}}{g}\right)\mathrm{Kwh}\right]}{\text{adsorption capacity}\;\left(\frac{\mathrm{mg}}{g}\right)\;X\;10^{-3}\frac{g}{\mathrm{mg}}}$$

The cost of preparing a sorbent and the operational cost of a treatment plant include the Total Capital Investment (TCI), which consists of the Working Capital Investment (WCC) and the Fixed Capital Estimation (Fce), as outlined in Equation (3) (Gopalakrishnan et al. 2020). The Fce covers all expenses related to equipment purchases, equipment installation, electrical systems, instrumentation and controls, buildings, construction, etc. Additionally, the WCC, which can account for up to 6.5% of the FCE, is also included. This cost can be calculated based on the volume of water to be treated, if the cost is expressed in dollars per cubic meter of treated water (Mahmoud et al. 2021). Further cost analyses can be performed using Equations (4) and (5).

(4) TCI=FCE+WCC

(5) $$\mathrm{AOC}=C_{\mathrm{RM}}+C_{\mathrm{UG}}+C_{U}+C_{G}$$

(6) $$C_{E}=\frac{\mathrm{AOC}}{E_{p}}$$

As outlined in Equation (4), the annual operating cost (AOC) for adsorbent synthesis can be determined by factoring in the cost of raw materials (CRM), the cost of managing waste generated during the process (CWG), the cost of utilities (CU), and any additional costs (CE). The cost per unit of the adsorbent synthesized (CE) can then be calculated by dividing the AOC by the annual adsorbent synthesized (EP), as shown in Equation (6) (Gopalakrishnan et al. 2020).

Across all treatment technologies, a core set of cross‐cutting metrics, including energy requirement (kWh/m^3^), chemical consumption (kg/m^3^), sludge or waste generation (kg/m^3^), and effluent compliance with discharge or reuse standards, should be routinely reported. Where possible, costs should be normalized to volumetric ($/m^3^) or mass‐based ($/kg COD or color removed) metrics, and the technology readiness level (laboratory, pilot, or full scale) should be clearly indicated. Adoption of such standardized reporting practices will enhance cross‐technology comparability and support the rational design of hybrid treatment trains. Embedding techno‐economic considerations at the experimental design stage is therefore essential to accelerate the translation of promising textile wastewater treatment technologies from laboratory research to industrial application.

## 10 Regulatory Compliance

In response to the persistent environmental risks posed by chemicals in textile wastewater, the International Dye Industry Wastewater Discharge Quality Standards have incorporated the Zero Discharge of Hazardous Chemicals (ZDHC) Program as a central framework guiding regulatory compliance within the textile and related chemical industries (Roy and Saha 2021). Although voluntary in nature, the ZDHC Wastewater Guidelines have become a de facto compliance reference across global textile supply chains, translating regulatory discharge requirements into actionable operational targets. The ZDHC Wastewater Guidelines establish stringent discharge limits for numerous hazardous substances commonly associated with textile manufacturing (Table S1). Although many treatment technologies reviewed in this study report removal efficiencies exceeding 80%–99%, percentage removal alone does not necessarily indicate compliance with ZDHC thresholds. Compliance depends on the final effluent concentration achieved, which is influenced by the initial contaminant concentration, wastewater matrix complexity, operational conditions, and treatment train configuration.

For conventional pollutants such as dyes, COD, color, suspended solids, and certain heavy metals, several technologies reviewed in this work, including adsorption, coagulation‐flocculation, membrane filtration, electrochemical oxidation, and AOPs, demonstrated removal efficiencies sufficient to potentially achieve ZDHC requirements when appropriately optimized. NF and RO are particularly promising due to their ability to simultaneously remove dyes, dissolved salts, and heavy metals at high efficiencies. However, compliance becomes considerably more challenging for contaminants with ultra‐trace discharge limits. For example, ZDHC limits for PFOS (0.01 μg/L), PFOA (1 μg/L), aromatic amines (0.1 μg/L), PAHs (1 μg/L), and phthalates (10 μg/L) require treatment systems capable of achieving near‐complete contaminant removal. Most studies reviewed evaluated these contaminants at mg/L concentrations and reported percentage removals rather than final residual concentrations, making direct assessment of ZDHC compliance difficult.

Overall, this literature survey suggests that achieving full compliance with ZDHC wastewater guidelines is unlikely through a single treatment technology alone. Instead, integrated treatment trains combining several processes that include advanced oxidation, adsorption, membrane polishing, and/or biological treatment are likely required to consistently achieve the stringent discharge limits established for both conventional pollutants and hazardous trace contaminants (Kumar et al. 2022). Future studies should therefore report final effluent concentrations relative to ZDHC thresholds and validate performance using real textile wastewaters under pilot‐ and full‐scale conditions to provide a more robust assessment of compliance potential.

## 11 Summary and Conclusion

As the textile industry continues to expand globally, the development of effective and scalable wastewater treatment technologies remains essential for reducing environmental pollution and protecting public health. This review evaluated the major physical, chemical, and biological treatment technologies currently applied to textile wastewater, including coagulation‐flocculation, membrane filtration, adsorption, advanced oxidation processes (AOPs), electrochemical treatment, and biological remediation.

Conventional processes such as coagulation–flocculation and biological treatment are best suited for pretreatment because they efficiently reduce suspended solids, color, and biodegradable organic matter. Membrane technologies, particularly UF, NF, and RO, are highly effective for polishing and water reuse applications, while ED‐based systems show promise for salt recovery. For persistent and emerging contaminants such as PFAS, PAHs, phthalates, UV absorbers, and aromatic amines, adsorption, and AOPs generally provide the highest removal efficiencies. Despite laboratory‐scale results with efficiencies exceeding 90% under laboratory conditions, several barriers limit industrial implementation. Adsorption processes require efficient sorbent regeneration, membrane systems are affected by fouling and concentrate management, photocatalytic processes face catalyst stability challenges, and electrochemical technologies remain energy‐intensive. In addition, most studies have been conducted using synthetic wastewater, limiting confidence in real‐world performance.

Future research should prioritize pilot‐ and full‐scale validation using real textile effluents, standardized performance reporting, long‐term stability assessments, and comprehensive techno‐economic and life‐cycle analyses. Overall, hybrid treatment systems that combine physicochemical/biological processes appear most promising for achieving regulatory compliance, water reuse, salt management, and effective removal of emerging contaminants cost‐effectively and sustainably.

## Author Contributions

**Adedapo O. Adeola:** conceptualization, investigation, funding acquisition, writing – original draft, visualization, validation, software, formal analysis, data curation, resources, writing – review and editing. **Stephanie Gora:** project administration, formal analysis, supervision, data curation, validation, writing – review and editing.

## Funding

The authors have nothing to report.

## Ethics Statement

This article does not contain any studies involving human or animal subjects.

## Conflicts of Interest

The authors declare no conflicts of interest.

## Supporting information


**Table S1:** ZDHC Wastewater limits for hazardous compounds in textile effluents (ZDHC) and possible treatment methods.
**Figure S1:** Adsorption isotherm and kinetic models and equations.

## Data Availability

Data sharing not applicable to this article as no datasets were generated or analysed during the current study.
