# Supplementary material for: Advances in Physicochemical and Biological Treatment of Textile Wastewater: An Applied Review of Methods, Technologies, and Costs
Source: Water Environ Res. 2026 Jul 18;98(7):e70490. doi: 10.1002/wer.70490 (PMC13379810; doi:10.1002/wer.70490)
Supplement: Supplementary file 1 — Table S1: ZDHC Wastewater limits for hazardous compounds in textile effluents (ZDHC) and possible treatment methods. Figure S1: Adsorption isotherm and kinetic models and equations. [file WER-98-e70490-s001.docx]

**Supplementary Files**

**Advances in Physicochemical and Biological Treatment of Textile Wastewater: An Applied Review of Methods, Technologies, and Costs**

**Table S1:** ZDHC Wastewater limits for hazardous compounds in textile effluents ([ZDHC](https://www.roadmaptozero.com/output%23guidelines)) and possible treatment methods.

| **Contaminants** | **Class of compounds** | **ZDHC limits (µg/L or ppb)** | **Potential treatment methods** | **References** |
| --- | --- | --- | --- | --- |
| Triclosan, o-Phenylphenol  Permethrin | Antimicrobials and Biocides | 100  500 | Adsorption using advanced nanomaterials or GAC | [1, 2] |
| Disperse yellow, red, blue, orange, brown | Dyes | 50 | Advanced oxidation process using O_3_/UV | [3] |
| Boric acid and related compounds containing boron.  Hexabromocyclodecane and other bromine containing organics. | Flame Retardants | 500  25 | Use of zero-valent iron (ZVI) coupled with microbial treatment | [4] |
| Perfluorooctane sulfonate (PFOS)  PFOA | Perfluorinated and Polyfluorinated Chemicals | 0.01  1 | Electrocoagulation, ozonation | [5, 6] |
| 1,2-benzenedicarboxylic acid, Bis(2-methoxyethyl)  phthalate, Butyl benzyl phthalate, Dinonyl phthalate, etc. | Phthalates | 10 | Bioremediation using *Cylindrotheca closterium* | [7] |
| Napthalene, phenanthrene, anthracene, pyrene, etc. | Polycyclic Aromatic Hydrocarbons | 1 | AOP processes and adsorption using advanced nanomaterials | [8, 9] |
| 2-naphthylamine, 2,4,5-trimethylaniline, 2,6-xylidine, etc | Restricted Aromatic Amines (Cleavable from Azo-Colourants) | 0.1 | AOP processes and adsorption using advanced nanomaterials | [10, 11] |
| 2-(2H-benzotriazol-2-yl)-4-(tert-butyl)-6-(secbutyl)  phenol (UV-350); 2,4-Di-tert-butyl-6-(5-chlorobenzotriazole-2-yl)  phenol (UV-327), etc. | UV Absorbers | 100 | AOP processes and adsorption using advanced nanomaterials | [12, 13] |
| Cresol, benzene, toluene, xylene, etc | Volatile Organic Compounds (VOC) | 1 | Granular Activated Carbon Adsorption | [14] |
| Chromium, arsenic, zinc, lead, cadmium, copper, cobalt, mercury | Heavy metals | 50, 50, 5000, 100, 100, 1000, 50, 10 | Adsorption and membrane processes | [15] |


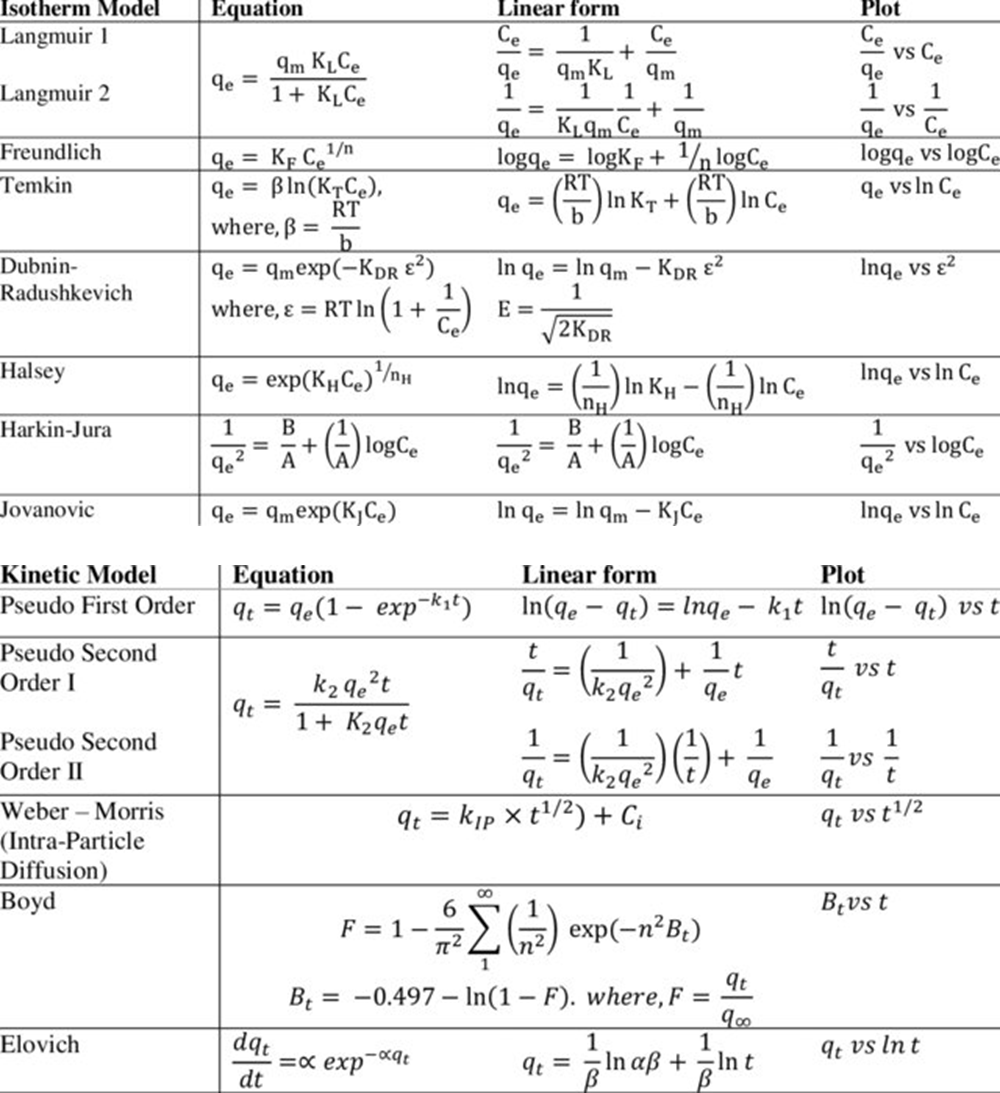


**Figure S1:** Adsorption isotherm and kinetic models and equations

References

[1] A. Katsigiannis, C. Noutsopoulos, J. Mantziaras, M. Gioldasi, Removal of emerging pollutants through Granular Activated Carbon, Chemical Engineering Journal 280 (2015) 49-57. <https://doi.org/https://doi.org/10.1016/j.cej.2015.05.109>.

[2] M. Alizadeh Fard, B. Barkdoll, Using recyclable magnetic carbon nanotube to remove micropollutants from aqueous solutions, Journal of Molecular Liquids 249 (2018) 193-202. <https://doi.org/https://doi.org/10.1016/j.molliq.2017.11.039>.

[3] Y. Dadban Shahamat, M. Masihpour, P. Borghei, S. Hoda Rahmati, Removal of azo red-60 dye by advanced oxidation process O3/UV from textile wastewaters using Box-Behnken design, Inorganic Chemistry Communications 143 (2022) 109785. <https://doi.org/https://doi.org/10.1016/j.inoche.2022.109785>.

[4] M. Kim, J. Han, Treatment techniques for removal of polybrominated diphenyl ethers (PBDEs) from real wastewater: Limitations, challenges, and future research directions, Journal of Water Process Engineering 63 (2024) 105463. <https://doi.org/https://doi.org/10.1016/j.jwpe.2024.105463>.

[5] T. Mu, M. Park, K.-Y. Kim, Energy-efficient removal of PFOA and PFOS in water using electrocoagulation with an air-cathode, Chemosphere 281 (2021) 130956. <https://doi.org/https://doi.org/10.1016/j.chemosphere.2021.130956>.

[6] A.Y. Lin, S.C. Panchangam, C.Y. Chang, P.K. Hong, H.F. Hsueh, Removal of perfluorooctanoic acid and perfluorooctane sulfonate via ozonation under alkaline condition, J Hazard Mater 243 (2012) 272-7. <https://doi.org/10.1016/j.jhazmat.2012.10.029>.

[7] J. Chi, Y. Li, J. Gao, Interaction between three marine microalgae and two phthalate acid esters, Ecotoxicol Environ Saf 170 (2019) 407-411. <https://doi.org/10.1016/j.ecoenv.2018.12.012>.

[8] G.K. Gaurav, T. Mehmood, M. Kumar, L. Cheng, K. Sathishkumar, A. Kumar, D. Yadav, Review on polycyclic aromatic hydrocarbons (PAHs) migration from wastewater, Journal of Contaminant Hydrology 236 (2021) 103715. <https://doi.org/https://doi.org/10.1016/j.jconhyd.2020.103715>.

[9] A.O. Adeola, B.A. Abiodun, D.O. Adenuga, P.N. Nomngongo, Adsorptive and photocatalytic remediation of hazardous organic chemical pollutants in aqueous medium: A review, Journal of Contaminant Hydrology 248 (2022) 104019. <https://doi.org/https://doi.org/10.1016/j.jconhyd.2022.104019>.

[10] A.O. Adeola, P.B.C. Forbes, Advanced Nanomaterials for Removal of Emerging Organic Pollutants From Water, in: G.L. Kyriakopoulos, M.G. Zamparas (Eds.), Novel Materials and Water Purification: Towards a Sustainable Future, Royal Society of Chemistry2024, p. 0. <https://doi.org/10.1039/9781837671663-00066>.

[11] N. Masomboon, C. Ratanatamskul, M.C. Lu, Chemical oxidation of 2,6-dimethylaniline in the fenton process, Environ Sci Technol 43(22) (2009) 8629-34. <https://doi.org/10.1021/es802274h>.

[12] W. Sun, H. Dong, Y. Wang, S. Duan, W. Ji, H. Huang, J. Gu, Z. Qiang, Ultraviolet (UV)-based advanced oxidation processes for micropollutant abatement in water treatment: Gains and problems, Journal of Environmental Chemical Engineering 11(5) (2023) 110425. <https://doi.org/https://doi.org/10.1016/j.jece.2023.110425>.

[13] J. Beljin, M. Kragulj Isakovski, T. Simetić, N. Đukanović, J. Molnar Jazić, S. Maletić, M. Vujić, Exploring the Adsorption Behavior of Organic UV Filter on Carbon-Based Materials as Potential Carriers of Organic Contaminants in the Aquatic Environment, Applied Sciences 14(20) (2024) 9424.

[14] X. Li, L. Zhang, Z. Yang, P. Wang, Y. Yan, J. Ran, Adsorption materials for volatile organic compounds (VOCs) and the key factors for VOCs adsorption process: A review, Separation and Purification Technology 235 (2020) 116213. <https://doi.org/https://doi.org/10.1016/j.seppur.2019.116213>.

[15] C.R. Fadila, M.H.D. Othman, M.R. Adam, R. Takagi, T. Yoshioka, W. Khongnakorn, M.A. Rahman, J. Jaafar, A.F. Ismail, Adsorptive membrane for heavy metal removal: Material, fabrication, and performance, Materials Today: Proceedings 65 (2022) 3037-3045. <https://doi.org/https://doi.org/10.1016/j.matpr.2022.03.582>.
